# Review of the millipede family Trichopolydesmidae in the Oriental realm (Diplopoda, Polydesmida), with descriptions of new genera and species

**DOI:** 10.3897/zookeys.414.7671

**Published:** 2014-06-05

**Authors:** Sergei I. Golovatch, Jean-Jacques Geoffroy, Didier VandenSpiegel

**Affiliations:** 1 Institute for Problems of Ecology and Evolution, Russian Academy of Sciences, Moscow, Russia; 2 Muséum national d’Histoire naturelle, Département Ecologie & Gestion de la Biodiversité, UMR 7204 CESCO, CNRS-MHNH-UPMC, Brunoy, France; 3 Musée Royal de l’Afrique centrale, Tervuren, Belgium

**Keywords:** Diplopoda, Trichopolydesmoidea, taxonomy, new genera, new species, key, Vietnam, Vanuatu

## Abstract

In the Oriental Region, the large, basically Northern Hemisphere family Trichopolydesmidae is shown to currently comprise 18 genera and 43 species. Based mainly on gonopod structure, all of them, as well as the whole family, are (re)diagnosed, including five new genera and seven new species. These new taxa are keyed, also being the first to be described from Indochina in general and from Vietnam in particular: *Aporodesmella* **gen. n.**, with three species: *A. securiformis* **sp. n.** (the type species), *A. similis* **sp. n.** and *A. tergalis* **sp. n.**, as well as the following four monotypic genera: *Deharvengius* **gen. n.**, with *D. bedosae* **sp. n.**, *Gonatodesmus* **gen. n.**, with *G. communicans* **sp. n.**, *Helicodesmus* **gen. n.**, with *H. anichkini* **sp. n.**, and *Monstrodesmus* **gen. n.**, with *M. flagellifer* **sp. n.** In addition, *Cocacolaria hauseri* Hoffman, 1987, hitherto known only from New Ireland Island, Papua New Guinea, is redescribed based on material from Vanuatu whence it is recorded for the first time. One of the new genera, *Gonatodesmus* **gen. n.**, provides a kind of transition or evolutionary bridge between Trichopolydesmidae and Opisotretidae, thus reinforcing the assignment of these two families to the single superfamily Trichopolydesmoidea.

## Introduction

The large, chiefly Northern Hemisphere millipede family Trichopolydesmidae has recently been rediagnosed and, together with the much smaller, Oriental and Papuan family Opisotretidae (Golovatch et al. 2013), shown to compose the superfamily Trichopolydesmoidea, suborder Polydesmidea (Golovatch 2013). The bulk of trichopolydesmid diversity, both generic and species, is taken up by what has until recently been considered as an independent pantropical family Fuhrmannodesmidae. Hoffman (1980, 1982) counted about 80 species from over 50 genera as unequivocally belonging to Fuhrmannodesmidae while another eight species in five genera were included in that family only provisionally.

Because the present contribution focuses on the fauna of the Oriental realm alone, by default all tropical and subtropical Asian, plus the few known Papuan trichopolydesmids have hitherto been treated as fuhrmannodesmids. However, following the recent synonymization of Fuhrmannodesmidae and several other, smaller, Holarctic, Nearctic or Palaearctic families with Trichopolydesmidae (Golovatch 2013), the names “fuhrmannodesmids” or “Fuhrmannodesmidae” will be referred to here in quotation marks.

Golovatch (1992, 1994), when sorting out the rich “fuhrmannodesmid” fauna of the Neotropical realm, suggested the following evolutionary scenario. He accepted as the basalmost those genera showing rather small, subglobose gonopod coxae that form no significant gonocoel in which to hinge the largely exposed, usually rather simple and elongate telopodites. Moreover, as in some Holarctic Trichopolydesmoidea, the prefemoral (= setose) part of the gonopod is mostly orientated transversely to the body axis, extending fully mesally across the coxae. Following a series of transitional states, such forms ultimately culminate in having the gonopod coxae strongly enlarged, forming a large gonocoel in which to conceal the clearly shortened, usually highly complex and deeply sunken telopodites. Their prefemoral parts already tend to be positioned increasingly parallel to the body’s main axis, thus providing a transition between the usually small-sized Trichopolydesmoidea (= so-called “micropolydesmoids”) to the normally medium- to large-sized Polydesmoidea (= so-called “macropolydesmoids”).

Naturally, similar general trends can be surmised to have occurred in the “fuhrmannodesmids” of the Afrotropical and, especially, Oriental realms, which also support fairly diverse faunas of this family. Prompted by the need to identify and allocate several species from Vietnam and Vanuatu, the present paper is an attempt to review all Oriental Trichopolydesmidae, i.e. 36 species or subspecies from 13 genera, arranged in alphabetic order:

1. *Assamodesmus lindbergi* Manfredi, 1955, the type species of *Assamodesmus* Manfredi, 1954, by original designation, described from the Himalayas of Assam, northeastern India (Manfredi 1955), redescribed by Golovatch (1988b).

2. *Coonoorophilus monstruosus* Carl, 1932, the type species of *Coonoorophilus* Carl, 1932, by monotypy, described from a single gynandromorph (abnormal, with only one gonopod retained) from southern India (Carl 1932).

3. *Hingstonia beatae* Golovatch, 1990, described from Nepal, Himalayas (Golovatch 1990).

4. *Hingstonia dorjulana* Golovatch, 1988, described from Bhutan, Himalayas (Golovatch 1988a).

5. *Hingstonia eremita* Carl, 1935, the type species of *Hingstonia* Carl, 1935, by monotypy, described from Nepal, Himalaya (Carl 1935), redescribed by Golovatch (1986).

6. *Hingstonia falcata* Golovatch, 1986, described from Nepal, Himalaya (Golovatch 1986).

7. *Hingstonia fittkaui* Golovatch, 1990, described from Nepal, Himalaya (Golovatch 1990).

8. *Hingstonia gogonana* Golovatch, 1988, described from Bhutan, Himalayas (Golovatch 1988a).

9. *Hingstonia pahakholana* Golovatch, 1990, described from Nepal, Himalayas (Golovatch 1990).

10. *Hingstonia pelelana* Golovatch, 1988, described from Bhutan, Himalaya (Golovatch 1988a).

11. *Hingstonia perarmata* Golovatch, 1986, described from Nepal, Himalaya (Golovatch 1986).

12. *Hingstonia serrata* Golovatch, 1987, described from Nepal, Himalayas (Golovatch 1987).

13. *Hingstonia sympatrica* Golovatch, 1990, described from Nepal, Himalayas (Golovatch 1990).

14. *Hingstonia variata* Golovatch, 1987, described from Nepal, Himalayas (Golovatch 1987), a little later recorded in Nepal again (Golovatch 1990).

15. *Hingstonia yeti* Golovatch, 1988, described from Bhutan, Himalaya (Golovatch 1988a).

16. *Kukkalodesmus exiguus* Carl, 1932, the type species of *Kukkalodesmus* Carl, 1932, by monotypy, described from southern India (Carl 1932).

17. *Magidesmus affinis* Golovatch, 1988, described from Bhutan, Himalayas (Golovatch 1988a).

18. *Magidesmus bhutanensis* Golovatch, 1988, the type species of *Magidesmus* Golovatch, 1988, by original designation, described from Bhutan, Himalayas (Golovatch 1988a).

19. *Mastodesmus zehntneri* Carl, 1911, the type species of *Mastodesmus* Carl, 1911, by monotypy, described from Java, Indonesia (Carl 1911).

20. *Nasodesmus cognatus* (Humbert, 1865), originally described as *Polydesmus cognatus* Humbert, 1865, from Sri Lanka (Humbert 1865), first made the type species of *Nasodesmus* Cook, 1896, by monotypy by Cook (1896), then redescribed and made the type species of *Lankadesmus* Carl, 1932, by original designation by Carl (1932). *Lankadesmus* is thus only a junior objective synonym of *Nasodesmus*.

21. *Ootacadesmus humilis* Carl, 1932, the type species of *Ootacadesmus* Carl, 1932, by original designation, described from southern India (Carl 1932).

22. *Peronorchus parvicollis* Attems, 1907, the type species of *Peronorchus* Attems, 1907, by monotypy, described from Buitenzorg (= Bogor), Java, Indonesia (Attems 1907), redescribed from material from Mauritius, Indian Ocean and assigned to the family Trichopolydesmidae (Mauriès and Geoffroy 1999), but recently transferred to “Fuhrmannodesmidae” (Golovatch et al. 2013).

23. *Pseudosphaeroparia cardamoni* Carl, 1932, described from southern India (Carl 1932).

24. *Pseudosphaeroparia cavernicola* Turk, 1945, described from a series of ♀ or juvenile syntypes from a cave near Dehra Dun, Uttar Pradesh, Himalayas of India (Turk 1945).

25. *Pseudosphaeroparia nilgirensis* Carl, 1932, described from southern India (Carl 1932).

26. *Pseudosphaeroparia palnensis* Carl, 1932, the type species of *Pseudosphaeroparia* Carl, 1932, by original designation, described from southern India (Carl 1932).

27. *Pseudosphaeroparia palnensis soror* Carl, 1932, described from southern India (Carl 1932).

28. *Sholaphilus albidus* Carl, 1932, the type species of *Sholaphilus* Carl, 1932, by monotypy (not by original designation, as mistakenly stated by Jeekel (1970)), described from southern India (Carl 1932).

29. *Sholaphilus asceticus* Golovatch, 1986, described from Nepal, Himalayas (Golovatch 1986).

30. *Sholaphilus dalai* Golovatch, 1986, described from Nepal, Himalayas (Golovatch 1986).

31. *Sholaphilus gompa* Golovatch, 1990, described from Nepal, Himalayas (Golovatch 1990).

32. *Sholaphilus lama* Golovatch, 1986, described from Nepal, Himalayas (Golovatch 1986).

33. *Sholaphilus martensi* Golovatch, 1986, described from Nepal, Himalayas (Golovatch 1986).

34. *Sholaphilus monachus* Golovatch, 1990, described from Nepal, Himalayas (Golovatch 1990).

35. *Topalodesmus communis* Golovatch, 1988, the type species of *Topalodesmus* Golovatch, 1988, by original designation, described from the Himalayas of Darjeeling District, northern India (Golovatch 1988b).

We can also add here one more genus and species from the region concerned:

36. *Cocacolaria hauseri* Hoffman, 1987, the type species of *Cocacolaria* Hoffman, 1987, by original designation, described from New Ireland, Papua New Guinea by Hoffman (1987). He refrained from assigning it to a family, but placed it close to or inside the Haplodesmidae. Here we consider *Cocacolaria* as a genus of Trichopolydesmidae on account of body and gonopod structure, especially, the shape of the collum and second tergite, these being drastically different from those observed in Haplodesmidae (Golovatch et al. 2009a, 2009b, 2010). Furthermore, below we provide the first record of this species in Vanuatu.

The following taxa must be excluded from Trichopolydesmidae or remain unclassified:

1. *Glenniea* Turk, 1945, with six species from the Himalayas of India, Nepal and Bhutan (Golovatch 1988a), as well as one species in Guangxi, southern China (Golovatch et al. 2012). A further two species, the first presumed troglobitic congeners, have been encountered in Sichuan, southern China (Golovatch & Geoffroy, in preparation). Hoffman (1980) listed this genus in Fuhrmannodesmidae, but Golovatch (1988a) transferred it to the Polydesmidae.

2. *Typhlopygmaeosoma hazeltonae* Turk, 1972, the type species of *Typhlopygmaeosoma* Turk, 1972, described from a cave near Shimla (formerly Simla), Himachal Pradesh, northern India (Turk 1972); first revised by Shear (1986) who equivocally assigned it to the family Opisotretidae, then by Golovatch (1988b) who placed it in the Polydesmidae.

In addition, closer unidentified “Fuhrmannodesmidae”, provisionally referred to as Gen. sp. 1, Gen. sp. 2 and Gen. sp. 3, have been recorded in the Cat Tien National Park, southern Vietnam (Golovatch et al. 2011). These species are described below.

As one can see from the above list, only one species, *Pseudosphaeroparia cavernicola*, is known too poorly (Turk 1945) to realistically become recognized. It is bound to remain enigmatic until a ♂ topotype has been obtained and properly described. In addition, *Coonoorophilus monstruosus* was based on a gynandromorph, thus strongly obscuring the identity both of the genus and species.

The main objective of the present paper is to address the generic classification of Trichopolydesmidae in the Oriental Region in order to identify and name a number of fresh samples from Vietnam and Melanesia.

**Abbreviations used:**

MNHN Muséum national d’Histoire naturelle, Paris, France

SEM Scanning electron microscopy

ZMUC Natural History Museum of Denmark, Copenhagen, Denmark

ZMUM Zoological Museum, State University of Moscow, Moscow, Russia

## Material and methods

Most of the material treated below was taken by Louis Deharveng, Anne Bedos (MNHN) and/or their collaborators in Vietnam. Several samples derive from ZMUM, collected by Alexander Anichkin (Joint Russia-Vietnam Tropical Center, Ho Chi Minh City, Vietnam) in a national park in southern Vietnam.

SEM micrographs were taken using a JEOL JSM-6480LV scanning electron microscope. After examination, SEM material was removed from stubs and returned to alcohol, all such samples being kept in MNHN.

The course of the seminal groove was always checked and, if necessary, depicted using transmission light microscopy.

### The main characters used in the classification of Trichopolydesmidae

The following characters have been used for defining genera in Oriental Trichopolydesmidae (= “Fuhrmannodesmidae”). Note that there is nothing particular in the peripheral, non-gonopod features that would uniquely characterize the Trichopolydesmidae (Golovatch 2011, Golovatch et al. 2013). The family-level differences tend to lie solely in gonopod conformation (see discussion below).

- **Number of body segments**

Like in most other families in Polydesmida, the number is 18, 19 or 20, largely being sex-characteristic. Thus, 20 segments in both sexes are known in most of the Oriental Trichopolydesmidae, 19 in both sexes only in *Assamodesmus*, *Peronorchus*, *Aporodesmella* gen. n., *Gonatodesmus* gen. n. and *Helicodesmus* gen. n., 19 in the ♂, but 20 in the ♀ in *Cocacolaria*, at least 19 in the ♂ of *Coonoorophilus* and *Ootacadesmus*. *Deharvengius* gen. n. is the sole genus in Trichopolydesmoidea which has only 18 segments in both sexes.

- **Metatergal setae**

All known species of Trichopolydesmidae show 3 transverse rows of tergal setae. The setation pattern is typical of the Polydesmidea, being 3+3 or more setae per row, only exceptionally 2+2 (*Deharvengius* gen. n.), plus 2 or 3 setae at each lateral margin. However, the setae themselves are typically ribbed, often helicoid, varying from long and sharp, like in *Cocacolaria*, *Mastodesmus* or *Deharvengius* gen. n., through short and simple, like in *Nasodesmus* or *Ootacadesmus*, to long and bacilliform, like in *Assamodesmus*, *Deharvengius* gen. n., *Helicodesmus* gen. n. or *Gonatodesmus* gen. n., or short and clavate, like in *Magidesmus*, *Pseudosphaeroparia*, *Sholaphilus* or some species of *Aporodesmella* gen. n. At least the bacilli- and claviform setae seem to always be modified, ribbed all along (e.g. Figs 11L, 13L, M, 16N, O). However, similar setae occur in some other families of Polydesmida such as Opisotretidae or Paradoxosomatidae (e.g. Golovatch et al. 2013).

- **Metatergal sculpture**

The pattern of metatergal sculpturing is typical of the Polydesmidea, i.e. 3 transverse rows of polygonal bosses or rounded tubercles, either with a more or less deep sulcus separating the first row from the two following ones or with clear sulci between all 3 rows. Each boss or tubercle is typically surmounted by a seta sometimes borne on a small knob. Among the Oriental Trichopolydesmidae, only very few species show truly distinct bosses or tubercles, e.g. *Cocacolaria* or *Mastodesmus*, whereas in the vast majority of cases the bosses are either flat or missing, and the transverse sulcus or sulci are largely superficial to wanting.

- **Location and size of ozopores**

Unlike species of Opisotretidae (Golovatch et al. 2013), Oriental Trichopolydesmidae usually show the ozopores small, opening flush on the dorsal surface and mostly located closer to the lateral margin of paraterga than to the caudal one. In *Cocacolaria* the ozopores are unusually prominent and, like in *Hingstonia*, *Mastodesmus* or *Nasodesmus*, placed just at or near the caudolateral paratergal corner. All other Oriental Trichopolydesmidae have the ozopores, albeit only rarely quite as large as in *Kukkalodesmus*, lying near the penultimate lateral incision of poriferous paraterga. So far as is known, the pore formula is always 5, 7, 9, 10, 12, 13, 15-17(18, 19), except in *Aporodesmella* gen. n., which is the sole genus in Trichopolydesmoidea which shows no ozopores whatever.

- **Shape and size of paraterga**

Variation in the degree of development of paraterga is considerable, ranging from fully wanting (some species of *Aporodesmella* gen. n.), through very poorly developed (nearly missing in *Cocacolaria*, *Peronorchus* or *Mastodesmus*) to evident or strong (*Nasodesmus*, *Magidesmus*, *Deharvengius* gen. n. or certain species of *Aporodesmella* gen. n.), usually varying between species in speciose genera. As in the other Polydesmida, paraterga tend to be slightly underdeveloped and set lower in ♀♀ and juveniles compared to ♂♂.

- **Antennae**

Normally, the antennae in Trichopolydesmoidea tend to be geniculate between antennomeres 5 and 6, these segments also usually bearing conspicuous apicodorsal groups of bacilliform sensilla. However, in a few Oriental Trichopolydesmidae, e.g. *Cocacolaria* or *Mastodesmus*, this geniculation is either feeble or totally wanting. Only exceptionally is ♂ antennomere 6 supplied with a conspicuous dorsoparabasal stump (some species of *Aporodesmella* gen. n.).

- ♂ **head modifications**

The ♂ vertex in Oriental Trichopolydesmidae, unlike that in some species from the Neotropical and Afrotropical realms (e.g. Golovatch 1994, Mauriès and Heymer 1996), usually remains unmodified. Only the ♂ of some species of *Aporodesmella* gen. n., as well as in *Gonatodesmus* gen. n. either shows a vertigial hump or the entire head in both sexes is clearly flattened dorsoventrally (*Deharvengius* gen. n.). Although the presence of such modifications is often considered as genus-characteristic (e.g. Mauriès and Heymer 1996), we believe they are only species-specific (e.g. Golovatch et al. 2013).

- **Legs**

Variation in leg length and armament in Oriental Trichopolydesmidae is quite pronounced, ranging from rather short and stout, sometimes also supplied with special ventral trichomes in the ♂, e.g. in some *Hingstonia*, to long and slender, e.g. in most of *Hingstonia* species, but the bulk of species show medium-sized, moderately to considerably stout legs which are usually devoid of modified trichomes in the ♂ and thus fail to differ much between the sexes. This contradicts Simonsen (1990) who stated that ♂ legs in “fuhrmannodesmids” show sphaerotrichomes. Only in *Kukkalodesmus*, *Nasodesmus* and *Ootacadesmus* does the ♂ have clearly inflated and elongated legs 3. Claw length seems to vary proportionately to leg length. In *Gonatodesmus* gen. n., ♂ tarsi 1 are supplied with modified, mostly bi- or trifid setae on the ventral side (Fig. 12B).

- **Gonopod structure**

In the systematics of any subgroup of the order Polydesmida, the gonopods offer most of the characters deemed useful, if not crucial, for the discrimination of genera and species. This fully applies to Trichopolydesmidae as well.

The gonopods in Trichopolydesmidae appear to vary much more strongly than in the Opisotretidae, the sole other family of Trichopolydesmoidea, in agreement with the fact that Trichopolydesmidae is much larger and is widespread throughout the Northern Hemisphere. Moreover, a gonopod-based diagnosis of Trichopolydesmidae is still not entirely satisfactory (Simonsen 1990, Golovatch 2011, 2013). This family appears to unite the micropolydesmoids in which the gonopod coxae are subglobose, from rather small to very large, while the prefemoral part is often orientated transversely to the body axis, usually extending fully mesally across the coxae. However, as the gonopod prefemora in trichopolydesmids grow shorter, the acropodites become orientated increasingly along the body axis, thus providing transitions to the superfamily Polydesmoidea (Golovatch et al. 2013).

Simonsen (1990), in his cladistic analysis of the suborder Polydesmidea, allotted the Opisotretidae the rank of an independent superfamily, Opisotretoidea. He also distinguished the “Fuhrmannodesmidae” from the other “families” of Trichopolydesmoidea through the presence of a separate tooth (= solenomere) terminating the seminal groove. Even if a solenomere is present in some other trichopolydesmoids, it was said its position would not be apical.

Such a definition is basically correct, although a few Neotropical “fuhrmannodesmids” show no solenomere whatsoever (Golovatch 1994). The lack both of an accessory seminal chamber and a hairy pulvillus is typically also correct, but this is characteristic of nearly all Trichopolydesmoidea except Opisotretidae (at least as opposed to the Polydesmoidea). What is definitely wrong in Simonsen’s (1990, p. 81) diagnosis of “Fuhrmannodesmidae” is the statement that the gonopod coxae, however large, fail to form a gonocoel, while the telopodite is devoid of a mesal branch (endomere). In contrast, according to Golovatch (1992, 1994), the degree of development of the gonocoxae (and their gonocoel) seems to be instrumental in properly assessing the main evolutionary trends in the family. In addition, the gonotelopodite in various Trichopolydesmidae is usually supplied with additional branches or other outgrowths, including mesal ones.

Amongst the Oriental Trichopolydesmidae, like in the Neotropical ones (Golovatch 1992, 1994), the genera in which the gonopod coxae and their gonocoel are still relatively small, leaving the telopodites strongly exposed, can be viewed as the basalmost. This condition is characteristic of *Assamodesmus*, *Cocacolaria* or *Mastodesmus*. In *Topalodesmus* and some *Sholaphilus*, the gonopod coxa has a strong frontal (= anterior) process which, like a rather deep gonocoel, is deemed to provide additional protection to the clearly exposed telopodites. Similar conditions have been noted in some Neotropical “Fuhrmannodesmidae” as well (Golovatch 1992, 1994). The deepest, maximally developed gonocoel, where the telopodites are extremely low, fully sunken and supplied with a tooth-shaped solenomere at the bottom of a mesal hollow, is found only in *Magidesmus*. Most of the remaining genera seem to represent intermediate states, whereas such species-rich genera as *Hingstonia*, *Pseudosphaeroparia* and *Sholaphilus* show a degree of intrageneric variation.

Only the gonopods of *Gonatodesmus* gen. n. stand apart in showing a kind of transition to Opisotretidae. Indeed, the gonocoel is small, the telopodites are strongly exposed and well separated, their basal parts are held parallel to the main axis, whereas their distal parts, following a clear midway geniculation, are directed abruptly laterad. Moreover, this genus demonstrates a strongly developed, hairy pulvillus and a small accessory seminal chamber so characteristic of most species of Opisotretidae (and Polydesmidae).

- **Vulva**

No special studies have been conducted on the conformation of the vulva in “Fuhrmannodesmidae”. Turk (1945) provided a crude sketch of an elongate and strongly setose vulva in *Pseudosphaeroparia cavernicola*. Mauriès and Heymer (1996) illustrated a rather short and poorly setose vulva of a *Sphaeroparia* species from Uganda, tropical Africa. The epigynal crest has never been described in any Trichopolydesmidae. Because these structures are too small and inconspicuous in the samples we have examined, they have been omitted from the descriptions.

## Generic reclassification

Based on the above information, as well as facing the need to properly allocate several new genera and species described below, we propose the following new diagnosis of Trichopolydesmidae and a new classification of the family’s constituent Oriental genera.

### Family Trichopolydesmidae

Verhoeff, 1910

http://species-id.net/wiki/Trichopolydesmidae

#### Diagnosis.

A family of the superfamily Trichopolydesmoidea, suborder Polydesmidea with 18, 19 or 20 segments, sometimes varying between sexes. Body very small to small (ca 2–20 mm long). Tegument microalveolate, limbus mostly microspiculate. ♂ head with or without vertigial modifications. Antennae often geniculate between segments 5 and 6, antennomeres 5 and 6 each usually with a compact group of bacilliform sensilla apicodorsally, rarely 6^th^ in ♂ with a dorsoparabasal stump. Metaterga usually with 3 regular, more rarely with more and/or irregular, transverse rows of sharp (= simple), bacilliform or clavate setae sometimes borne on knobs; side margin of paraterga incised or tuberculate, with 2 or 3 setae. Paraterga from absent to strongly developed, usually only slightly indented laterally, only exceptionally deeply trilobate (*Trilobodesmus* Golovatch & Mauriès, 2007). Pore formula usually normal: 5, 7, 9, 10, 12, 13, 15–17 (18,19), but sometimes ozopores completely wanting, rarely unusually large, normally opening flush on dorsal or dorsolateral surface either near penultimate lateral incision or at caudal corner of paraterga. Legs rather short to long, ♂ ones often stouter and elongate, rarely modified, inflated, only sometimes with peculiar ventral setae, including sphaerotrichomes.

Gonopods with subglobose, medially fused coxae, these being rather small to quite large (with correspondingly large gonocoel), micropapillate and at most only slightly setose laterally, sometimes with a frontal (= anterior) process, each supporting a cannula medially (exception: *Caucasodesmus* Golovatch, 1987, in which there is no cannula) and a sack-shaped to elongate telopodite. The latter ranging from stout and short, deeply sunken inside a deep gonocoel, to slender and long, nearly fully exposed, strongly to modestly curved caudad or mesad, only exceptionally geniculate (distal half directed abruptly laterad: *Gonatodesmus* gen. n.) or perforating coxal wall with a prominent process (*Schizotelopus* Verhoeff, 1941), usually complex, with various processes or outgrowths, sometimes fringed; seminal groove normally terminating distally or apically on a separate branch or tooth (= solenomere), rarely on a tooth inside gonocoel, exceptionally absent (*Caucasodesmus*). Typically neither an accessory seminal chamber nor a hairy pulvillus (a few exceptions, e.g. *Gonatodesmus* gen. n.).

#### Type genus.

*Trichopolydesmus* Verhoeff, 1910.

#### Remarks.

Most of the above somatic and gonopod features of Trichopolydesmidae are in no way unique to the family, sometimes being also encountered, in various combinations, in the micropolydesmoid family Opisotretidae of the same superfamily Trichopolydesmoidea, as well as in certain macropolydesmoid members of the family Polydesmidae, superfamily Polydesmoidea (Golovatch et al. 2013, Golovatch 2013). The finely microspiculate limbus is also characteristic of most of the Polydesmidea, and is another feature whose importance was obviously overestimated by Simonsen (1990). It is only the gonopod structure that seems to be characteristic of Trichopolydesmidae, at least so to a certain extent. Superficially, female and/or juvenile trichopolydesmids from the Oriental realm are not or only barely distinguishable from the often sympatric or even syntopic female or juvenile Opisotretidae or smaller Polydesmidae.

The following 13 nominate Oriental genera of Trichopolydesmidae seem to be valid, and are arranged and defined below in alphabetic order.

### Assamodesmus

Manfredi, 1954

http://species-id.net/wiki/Assamodesmus

#### Diagnosis.

19 segments (♂, ♀), pore formula normal, ozopores placed closer to lateral than to caudal margin of paratergite; ♂ vertex unmodified, paraterga modest, 3 rows of bacilliform metatergal setae; gonopod telopodites subfalcate and clearly twisted, held parallel to main body axis, strongly exposed (gonocoel small); solenomere (**sl**) a long, simple, distal spine with an adjacent, distal, bipartite solenophore (**sph**) consisting of an apical spine and a massive velum.

#### Type species.

*Assamodesmus lindbergi* Manfredi, 1955, by original designation.

#### Remarks.

This well-defined Himalayan genus remains monobasic.

### Cocacolaria

Hoffman, 1987

http://species-id.net/wiki/Cocacolaria

#### Diagnosis.

19 (♂) or 20 (♀) segments, pore formula normal, ozopores placed closer to lateral than to caudal margin of paratergite; ♂ vertex unmodified, paraterga poorly developed, 3 rows of sharp long metatergal setae, metatergal sculpture (bosses) unusually high; gonopod telopodites subfalcate, crossing mesally, very strongly exposed (gonocoel small); **sl** a small branchlet at midway along telopodite; **sph** distal to **sl** unipartite, large, bifid and rather simple.

#### Type species.

*Cocacolaria hauseri* Hoffman, 1987, by original designation.

#### Remarks.

This genus remains monobasic, its sole constituent species being quite widespread in Melanesia. *Cocacolaria hauseri* is redescribed below, based on samples from Vanuatu.

### Coonoorophilus

Carl, 1932

http://species-id.net/wiki/Coonoorophilus

#### Diagnosis.

19 segments (♂), ♀ unknown, much like *Ootacadesmus* (see below), but gonopods strongly resembling those of *Sholaphilus*.

#### Type species.

*Coonoorophilus monstruosus* Carl, 1932, by monotypy.

#### Remarks.

This monobasic genus is retained only provisionally, because it combines the basic characters of *Ootacadesmus*, *Kukkalodesmus*, *Pseudosphaeroparia* and *Sholaphilus*. The gynandromorph ♂ holotype of *Coonoorophilus monstruosus* retained only a single gonopod.

### Hingstonia

Carl, 1935

http://species-id.net/wiki/Hingstonia

#### Diagnosis.

20 segments (♂, ♀); pore formula normal, ozopores lying opposite 3^rd^ incision, placed closer to lateral than to caudal margin of paratergite; ♂ vertex unmodified, paraterga keel-shaped, rather well developed, 3 rows of short, bacilliform to clavate setae on knobs; ♂ legs nearly normal, often with sphaerotrichomes; gonopod coxae from modest to large (gonocoel also from modest to deep), telopodites strongly exposed, usually massive, never fringed; **sph**(distal part of telopodite) complex, at least with one process or outgrowth, usually more; **sl** a rather long, simple, apical or subapical branch.

#### Type species.

*Hingstonia eremita* Carl, 1935, by monotypy.

#### Remarks.

This relatively species-rich, well-defined, Himalayan genus mostly contains relatively large species (up to 20 mm long).

### Kukkalodesmus

Carl, 1932

http://species-id.net/wiki/Kukkalodesmus

#### Diagnosis.

20 segments (♂, ♀); pore formula normal, ozopores lying opposite 2^nd^ incision, placed closer to lateral than to caudal margin of paratergite; ♂ vertex unmodified, paraterga keel-shaped, rather well developed, 3 rows of short, bacilliform to clavate metatergal setae on knobs; ♂ femur 3 strongly enlarged; gonopod coxae not very large (gonocoel modest), telopodites strongly exposed; **sph** distal, complex, with 2 caudal lobes and a frontal process, **sl** a small, plumose, subapical branchlet.

#### Type species.

*Kukkalodesmus exiguus* Carl, 1932, by monotypy.

#### Remarks.

This monotypic genus seems to be rather poorly defined versus *Sholaphilus*, *Pseudosphaeroparia*, *Coonoorophilus* and *Ootacadesmus*, especially given the strong variation in gonopod structure in the relatively species-rich genera *Sholaphilus* and *Pseudosphaeroparia* (see also above and below).

### Magidesmus

Golovatch, 1988

http://species-id.net/wiki/Magidesmus

#### Diagnosis.

20 segments (♂), ♀ unknown; pore formula normal, ozopores placed closer to lateral than to caudal margin of paratergite; ♂ vertex unmodified, paraterga well developed and set quite high, 3 rows of short clavate metatergal setae; gonopod coxae very large (gonocoel deep), telopodites deeply sunken and barely exposed, sac-shaped, hollow in the middle, apically somewhat fringed; a **sph** missing, **sl** a small denticle at bottom of this hollow.

#### Type species.

*Magidesmus bhutanensis* Golovatch, 1988, by original designation.

#### Remarks.

This small Himalayan genus is well-defined.

### Mastodesmus

Carl, 1911

http://species-id.net/wiki/Mastodesmus

#### Diagnosis.

20 segments (♂, ♀); pore formula normal, ozopores placed closer to lateral than to caudal margin of paratergite; ♂ vertex unmodified, paraterga almost missing and set low, 3 rows of long, curved, simple metatergal setae on unusually prominent tubercles; gonopod coxae modest (gonocoel not very deep), telopodites bipartite in distal half, well exposed, very simple; **sph** a simple exomere branch, **sl** a long, simple, caudal spine branching off from distal half of telopodite.

#### Type species.

*Mastodesmus zehntneri* Carl, 1911, by monotypy.

#### Remarks.

This monobasic genus strongly resembles *Peronorchus* and *Nasodesmus*, but seems to be sufficiently well defined.

### Nasodesmus

Cook, 1896

http://species-id.net/wiki/Nasodesmus

#### Diagnosis.

20 segments (♂, ♀); pore formula normal, ozopores placed closer to lateral than to caudal margin of paratergite; ♂ vertex unmodified, paraterga well developed, 3 rows of short simple metatergal setae; ♂ legs 3 strongly elongate, the femur very strongly enlarged; gonopod coxae not very large (gonocoel not too deep); telopodites strongly exposed, simple, unipartite, slightly curved mesad; **sph** (exomere) simple, strong, with a small process at base; **sl** a small, subapical, mesal branchlet.

#### Type species.

*Polydesmus cognatus* Humbert, 1865, by original designation.

#### Remarks.

This monobasic genus strongly resembles *Peronorchus* and *Mastodesmus*, but seems to be sufficiently well defined.

### Ootacadesmus

Carl, 1932

http://species-id.net/wiki/Ootacadesmus

#### Diagnosis.

19 segments (♂), ♀ unknown; pore formula normal, ozopores placed closer to lateral than to caudal margin of paratergite; ♂ vertex unmodified, paraterga well developed, 3 rows of very short and simple metatergal setae; ♂ legs 3 clearly elongate, the femur very strongly enlarged; gonopod coxae very large (gonocoel deep); telopodites deeply sunken and only very little exposed, with a parabasal process; **sph** bipartite, with various apical outgrowths, including a lateral velum, **sl** a small subapical branchlet.

#### Type species.

*Ootacadesmus humilis* Carl, 1932, by original designation.

#### Remarks.

This monotypic genus seems to be rather poorly defined versus *Sholaphilus*, *Pseudosphaeroparia*, *Coonoorophilus* and *Kukkalodesmus* (see also above and below).

### Peronorchus

Attems, 1907

http://species-id.net/wiki/Peronorchus

#### Diagnosis.

19 segments (♂, ♀); pore formula normal, ozopores placed closer to lateral than to caudal margin of paratergite; ♂ vertex unmodified; paraterga very small, body nearly cylindrical, 3 rows of 3+3 or 4+4, rather long bacilliform metatergal setae (regardless of lateral setae on paraterga); gonopod coxae with gonocoel not deep; telopodites clearly exposed, without parabasal processes, moderately curved and held parallel to each other; **sph** bifid distally, with **sl** a rather long, flagelliform, apical branch lying in **sph** fork.

#### Type species.

*Peronorchus parvicollis* Attems, 1907, by monotypy.

#### Remarks.

This genus strongly resembles *Nasodesmus* and *Mastodesmus*, but seems to be sufficiently well defined.

### Pseudosphaeroparia

Carl, 1932

http://species-id.net/wiki/Pseudosphaeroparia

#### Diagnosis.

20 segments (♂, ♀); pore formula normal, ozopores placed closer to lateral than to caudal margin of paratergite; ♂ vertex unmodified, paraterga modest to well developed, 3 rows of short clavate metatergal setae; gonopod coxae very large (gonocoel deep) to modest; telopodites from rather deeply sunken to rather clearly exposed, with or without parabasal processes; **sph** unipartite, with various outgrowths, **sl** a rather evident apical branch.

#### Type species.

*Pseudosphaeroparia palnensis* Carl, 1932, by original designation.

#### Remarks.

This small genus is quite poorly defined versus *Sholaphilus*, *Coonoorophilus*, *Ootacadesmus* and *Kukkalodesmus* (see also above and below).

### Sholaphilus

Carl, 1932

http://species-id.net/wiki/Sholaphilus

#### Diagnosis.

20 segments (♂, ♀); pore formula normal, ozopores placed closer to lateral than to caudal margin of paratergite; ♂ vertex unmodified, paraterga modest to well developed, 3 rows of short clavate metatergal setae; gonopod coxae very large (gonocoel deep) to modest, sometimes with a strong frontal process; telopodites rather deeply sunken and usually only little exposed, but sometimes rather strongly exposed, with or without parabasal processes; **sph** uni- to only faintly bipartite, with various outgrowths, densely fringed, **sl** a small apical or subapical tooth or branchlet.

#### Type species.

*Sholaphilus albidus* Carl, 1932, by monotypy.

#### Remarks.

Even though this rather small genus is quite poorly defined versus *Ootacadesmus*, *Pseudosphaeroparia*, *Coonoorophilus* and *Kukkalodesmus* (see above), several species of *Sholaphilus* from the Himalayas have been described.

### Topalodesmus

Golovatch, 1988

http://species-id.net/wiki/Topalodesmus

#### Diagnosis.

20 segments (♂, ♀); pore formula normal, ozopores placed closer to lateral than to caudal margin of paratergite; ♂ vertex unmodified, paraterga modest, 3 rows of short simple/claviform metatergal setae; gonopod coxae massive, each with a prominent frontal process; gonocoel rather deep, but telopodites subfalcate and strongly exposed, tripartite, each with a strong, parabasal, unciform, caudomedial process, a short laterobasal stump-shaped exomere, and a prominent, elongate **sph** crowned with a small apical **sl**.

#### Type species.

*Topalodesmus communis* Golovatch, 1988, by original designation.

#### Remarks.

This monobasic Himalayan genus is well-defined (see also below under *Monstrodesmus* gen. n.).

### Descriptions

#### Cocacolaria hauseri

Hoffman, 1987

http://species-id.net/wiki/Cocacolaria_hauseri

Figs 1 , 2

Cocacolaria hauseri Hoffman, 1987: 38.

##### Material examined.

1 ♂, 7 ♀, 1 fragment (MNHN JC 354), Vanuatu, Espirito Santo Island, Natawa forest, small doline Arifos, 167.183167E, 15.2962S, sieved litter & Berlese extraction, 24.09.2006, leg. L. Deharveng & A. Bedos (SK06-24-24); 1 ♂ (SEM), same locality, date and collectors (SK06-24-22); 3 ♀ (MNHN JC 354), Espirito Santo Island, Boutmas, forest near entrance to Cave Fapon, 166.9648833E, 15.33101667S, 380 m a.s.l., litter, Berlese extraction, 08.09.2006, leg. L. Deharveng & C. Rahmadi (SK06-08-24).

##### Redescription.

Length of adults of both sexes 4–6 mm (♂, ♀), width of midbody pro- and metazonae ca 0.3 and 0.4 mm, respectively. Coloration in alcohol from uniformly pallid to light yellowish.

**Figure 1.**
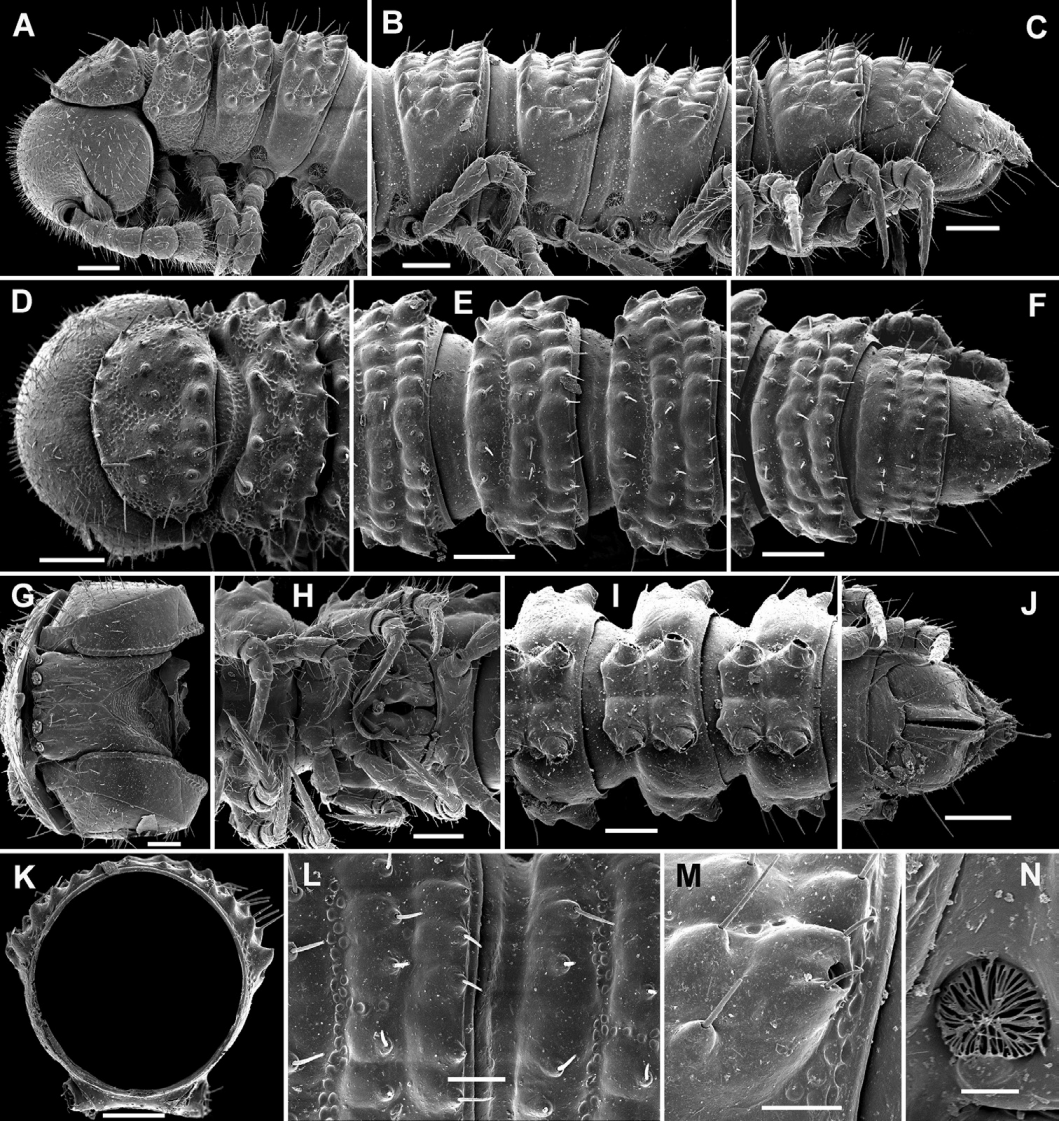
*Cocacolaria hauseri* Hoffman, 1987, ♂ from Natawa forest, Espirito Santo Island, Vanuatu; **A, D** anterior part of body, lateral and dorsal views, respectively **B, E, I** midbody segments, lateral, dorsal and ventral views, respectively **C, F, J** posterior part of body, lateral, dorsal and ventral views, respectively **G** head, ventral view **H** body segments 6 and 7, ventral view **K** cross-section of a midbody segment, caudal view **L** midbody tergal sculpture and setae, dorsal view **M** caudal part of a midbody pore-bearing paratergite, lateral view **N** midbody spiracle. Scale bars: **A–F, H–K** 0.1 mm; **G, L, M** 0.05 mm; **N** 0.02 mm.

Body with 19 (♂) or 20 (♀) segments. Tegument mainly dull, at most slightly shining, texture very delicately alveolate. Head densely pilose throughout; epicranial suture superficial and thin (Fig. 1A); isthmus between antennae about 1.5 times as broad as diameter of antennal socket. Antennae short, reaching only behind (♂) or midway of collum (♀) when stretched dorsally, not geniculate, very clearly clavate, especially so due to largest antennomere 6, the latter with a small, but evident distodorsal group of bacilliform sensilla.

Body moniliform, especially so in ♂ (Fig. 1D–F). In width, collum << head < segment 2 < 3 = 4 < 5(6) = 15 (♂, ♀), thereafter body gradually tapering towards telson. Paraterga very poorly developed, mainly represented by setigerous tubercles (Fig. 1A–F, H–K), starting from collum, set rather low (at about upper 1/3–1/2 of segment’s height), leaving a highly convex dorsum (Fig. 1A–C & K). Caudal corner of postcollum paraterga increasingly tuberculiform, mainly clearly rounded, not extending behind rear tergal margin. Pore formula normal; ozopores evident, round, lying dorsolaterally on top of caudolateral tubercle of paraterga very close to lateral margin, more remote from caudal margin (Fig. 1M). Collum subovoid, each following metatergum with 3 transverse rows of mostly long, pointed, very faintly ribbed, helicoid setae largely borne on 6+6 unusually high tubercles, these being especially prominent in last row (Fig. 1A–F). Stricture between pro- and metazonae deep, rather narrow and smooth. Limbus thin and entire, not microdenticulate. Pleurosternal carinae absent (Fig. 1A). Epiproct short, conical, directed caudoventrally; pre-apical papillae evident (Fig. 1C, F, J). Hypoproct nearly semi-circular (Fig. 1J), setiferous papillae at caudal corners very clear, stalk-shaped and well separated.

**Figure 2.**
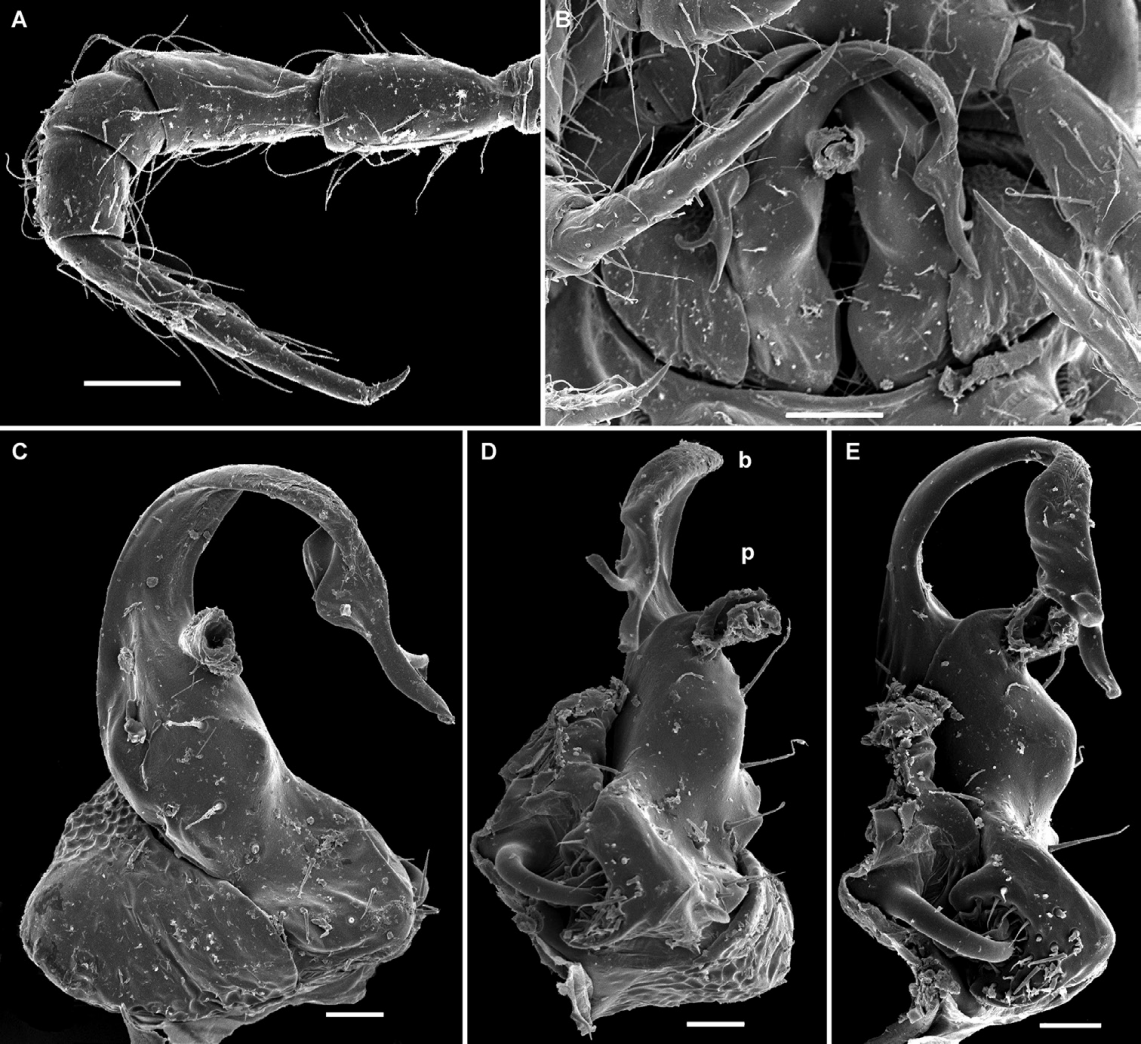
*Cocacolaria hauseri* Hoffman, 1987, ♂ from Natawa forest, Espirito Santo Island, Vanuatu; **A** midbody leg, lateral view **B** gonopod aperture with both gonopods *in situ*, ventral view **C** right gonopod, lateral view **D, E** left gonopod, caudomesal and mesal views, respectively. Scale bars: **A, B** 0.05 mm; **C–E** 0.02 mm. Designations of gonopod structures in text.

Sterna without modifications, broad, flat, poorly setose (Fig. 1H–J). Stigma openings flat, beset with dense and long filaments (Fig. 1N). Legs in ♀ and juveniles clearly shorter and slenderer, ca 1.0–1.1 times as long as body height, in ♂ somewhat incrassate and ca 1.3–1.4 times as long as body height (Figs 1B, 2A); tarsi longest; sphaerotrichomes missing (Fig. 2A). Each ♂ coxa 2 with a short, cylindrical, distoventral gonapophysis.

Gonopod aperture transversely oblong-oval, taking up most of ventral part of metazonite 7, its edges nowhere elevated (Figs 1H, 2B). Gonopods (Fig. 2B–E) with rather moderately large, globose, bare, ventrolaterally papillate, medially fused coxae carrying normal cannulae mesally. Telopodite strongly elongate and curved, subunciform, bipartite, directed mesad and crossing *in situ*, each at about midway with a short, mesal, calyciform process (**p**) (= solenomere) terminating a fully mesal seminal groove, with neither an accessory seminal chamber nor a hairy pulvillus. Main branch (**b**) (= solenophore) bifid and slightly expanded subterminally.

##### Remarks.

This species, originally described from a single ♂ taken in a cave in Lelet Plateau, New Ireland, Papua New Guinea (Hoffman 1987), appears to be common and widespread in Vanuatu. However, among the literally hundreds of specimens taken on Espiritu Santo and Malo islands by the famous SANTO 2006 expedition, held by the MNHN (Bouchet et al. 2009, 2011, 2012), there were only two adult males. This might be evidence of seasonality or partial parthenogenesis, or both.

The original description is quite detailed and nicely illustrated (Hoffman 1987) and we are certain about the identity of our Vanuatu samples. We provide SEM images (Figs 1 and 2) to document the identity and to complement Hoffman’s (1987) description.

#### Aporodesmella gen. n.

http://zoobank.org/3B37A67E-1F19-46E5-985C-E9AF3BD33826

http://species-id.net/wiki/Aporodesmella

##### Diagnosis.

19 segments (♂, ♀); ozopores wanting; ♂ vertex modified or not; ♂ antennomere 6 sometimes with a conspicuous dorsoparabasal stump; paraterga absent to rather well developed, 3 rows of 3+3 or 4+4, short or long, bacilli- and/or claviform metatergal setae (regardless of lateral setae on or instead of paraterga); gonopod coxae with gonocoel not deep; telopodites clearly exposed, mostly or considerably represented by elongate prefemoral parts, at most only moderately curved, medially subcontiguous and held parallel to each other; acropodites, or solenophores (**sph**), lying distal to prefemoral parts shortened, only sometimes set off basally by a ventral sulcus, subtruncate, usually more or less coaxial with prefemoral parts, but sometimes extended laterad into a distinct process; solenomere (**sl**) subapical, rather long to vestigial.

##### Name.

To emphasize the lack of ozopores, feminine.

##### Type species.

*Aporodesmella securiformis* sp. n., by present designation.

##### Remarks.

The new genus is unique among the Trichopolydesmoidea for its complete loss of ozopores. Among Oriental Trichopolydesmidae, *Aporodesmella* gen. n. seems to be especially similar to *Peronorchus*, sharing with it not only 19 body segments in both sexes, but also the gonopods demonstrating a modest gonocoel, elongate, clearly exposed and modestly curved telopodites which are (sub)contiguous medially and held parallel to each other (and to the main body axis), the solenophore being enlarged, more or less cup-shaped and membranous while the solenomere is a short subapical process or tooth. However, the prefemoral (= densely setose) part of the gonopod in *Peronorchus* is clearly shorter.

The following three new species are attributed to *Aporodesmella* gen. n.

#### Aporodesmella securiformis sp. n.

http://zoobank.org/A08E5C4F-B6E8-493B-A832-6C383E1FC438

http://species-id.net/wiki/Aporodesmella_securiformis

Figs 3 , 4

##### Type material.

Holotype ♂ (MNHN JC 355), Vietnam, Kien Giang Province, Kien Luong, Hon Chong, Nui Bai Voi, 104.618E, 10.218N, soil, Berlese extraction, 02.06.2008, leg. L. Deharveng & A. Bedos (Vn08-045).

Paratypes: 1 ♂, 4 ♀ (MNHN JC 355), 1 ♂, 1 ♀ (ZMUM ρ2346), 1 ♂ (SEM), same locality, together with holotype; 2 ♂, 1 ♀ (MNHN JC 355), 1 ♂ (SEM), same province, Kien Luong, Hon Chong, Nui Hang Tien, litter & soil, Berlese extraction, 02.06.2008, leg. Ly Ngoc Sam (Vn08-065); 3 ♂ (MNHN JC 355), 1 ♂ (SEM), same data (Vn08-066).

##### Name.

To emphasize the axe-shaped solenophore.

##### Diagnosis.

Differs from congeners except *Aporodesmella similis* sp. n. by the presence of a remarkable dorsoparabasal stump on ♂ antennomere 6, from *Aporodesmella similis* sp. n. and other congeners by a peculiar, unusually short, axe-shaped solenophore and a simple, lanceolate, shorter and stout solenomere.

##### Description.

Length of adults ca 2.8–2.9 mm (♂, ♀), up to 3.0 mm (♀), width of midbody pro- and metazonae 0.15 and 0.2 (♂, ♀), up to 0.2 and 0.25 mm (♀), respectively. Coloration in alcohol uniformly pallid, tegument translucent.

**Figure 3.**
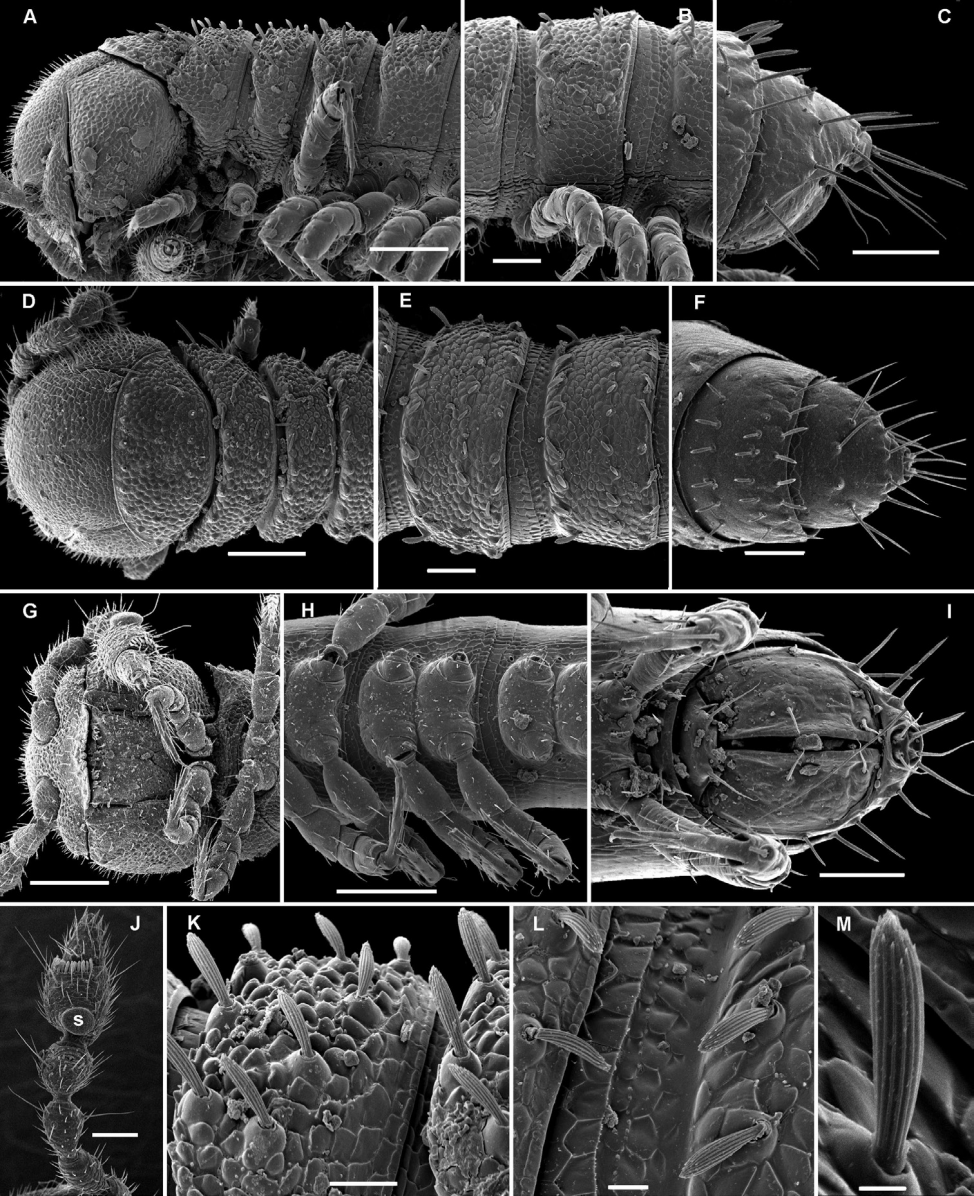
*Aporodesmella securiformis* sp. n., ♂ paratype from Nui Hang Tien; **A, D, G** anterior part of body, lateral, dorsal and ventral views, respectively **B, E, H** midbody segments, lateral, dorsal and ventral views, respectively **C, F, I** posterior part of body, lateral, dorsal and ventral views, respectively **J** antenna, dorsal view **K** tergal texture and setae, lateral view **L** tergal setae, limbus and stricture region, sublateral view **M** tergal seta, lateral view. Scale bars: **A, D, H** 0.1 mm; **B, C, E–G, I, J** 0.05 mm; **K** 0.02 mm; **L** 0.01 mm; **M** 0.005 mm. Designation of antennal structure in text.

**Figure 4.**
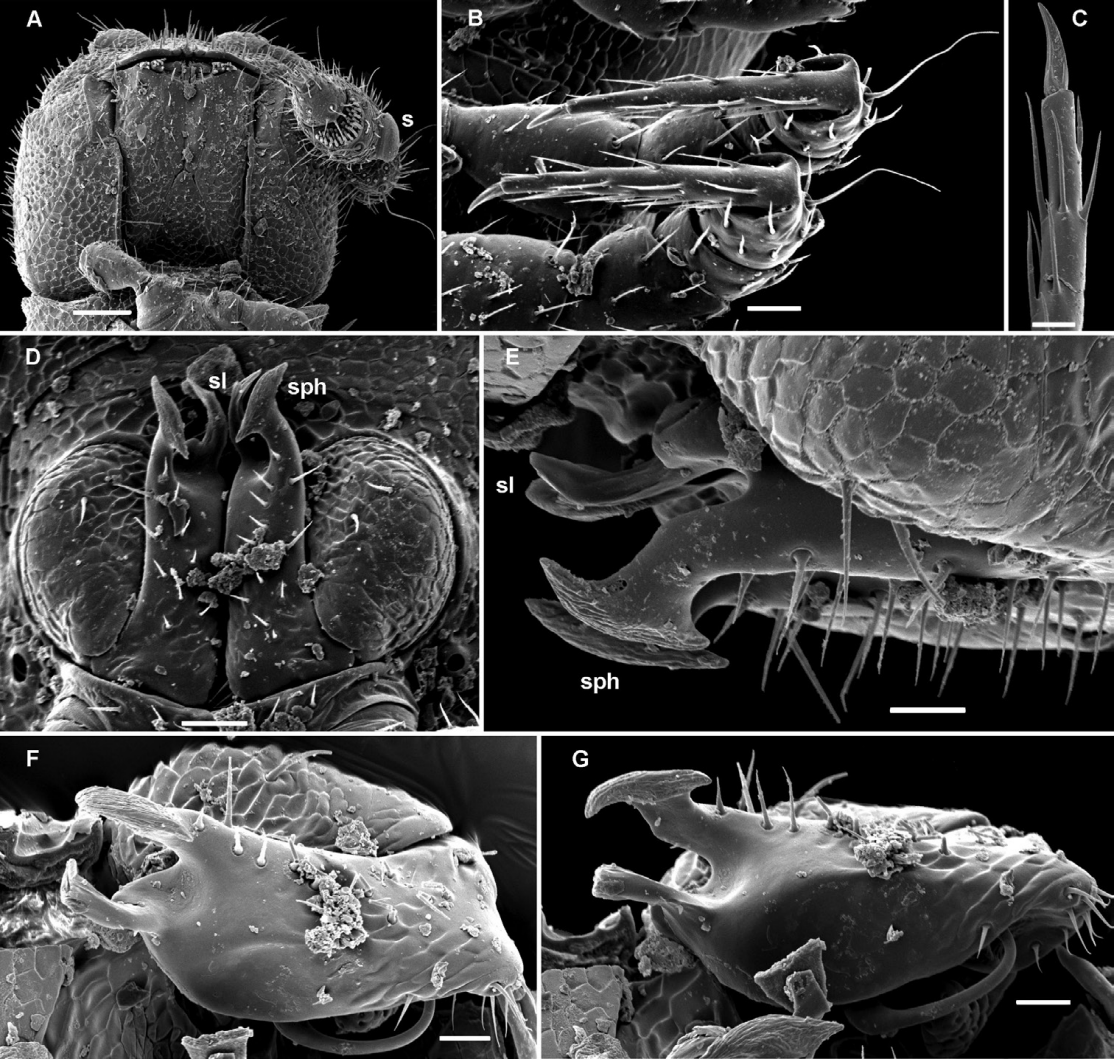
*Aporodesmella securiformis* sp. n., ♂ paratype from Nui Hang Tien; **A** head, ventral view **B** midbody legs, ventral view **C** midbody tarsus and claw, lateral view **D, E** gonopod aperture and gonopods *in situ*, ventral and lateral views, respectively **F, G** left gonopod, subventral and ventromedial views, respectively. Scale bars: **A** 0.05 mm; **B, D** 0.02 mm; **C, E–J** 0.01 mm. Designations of gonopod structures in text.

Body moniliform, with 19 segments (♂, ♀). Tegument mainly dull, at most slightly shining, texture very delicately alveolate and microgranulate. Head without modifications, densely pilose throughout except occiput; epicranial suture superficial and thin; genae squarish (Fig. 3A, D, G); gnathochilarium narrow, setae dense and short (Fig. 4A); isthmus between antennae about as broad as diameter of antennal socket (Fig. 3G). Antennae very short, reaching only behind collum (♂, ♀) when stretched dorsally, not geniculate, strongly clavate due to an abruptly and particularly enlarged antennomere 6, the latter with a usual, tight, distodorsal group of numerous bacilliform sensilla, but in ♂ also with a large, highly conspicuous, dorsoparabasal, rounded stump (**s**); antennomere 5 with a loose distodorsal group of only a few tiny sensilla, 7^th^ with a tiny mid-dorsal knob (Figs 3G, J, 4A).

In width, head > segment 2 > collum = segment 3 = 4 > 5(6) = 16 (♂, ♀), thereafter body gradually tapering towards telson (Fig. 3D–F). Paraterga wanting, metazonae subcylindrical, dorsum strongly convex (Fig. 3A–I). Ozopores totally absent (Fig. 3A, B, D–F, K). Collum subovoid, each following metatergum mostly with 4+4 rather long, slightly blunted, subclavate to subbacilliform, thickened and longitudinally ribbed setae arranged in 3 transverse regular rows and borne on minute knobs (Fig. 3A–F, K–M). Stricture between pro- and metazonae rather wide and shallow, scaly like rear part of prozonae. Limbus very fine and microcrenulate (Fig. 3L). Pleurosternal carinae thin lines (Fig. 3A, B, H). Epiproct short, conical, directed caudoventrally; pre-apical papillae small (Fig. 3C, F, I). Hypoproct subtrapeziform, caudal setigerous papillae evident and well separated (Fig. 3I).

Sterna without modifications, rather broad and sparsely setose (Fig. 3H). Legs short, ca 1.3–1.4 (♂) or 1.1–1.2 times as long as midbody height (♀); ♂ prefemora, femora, postfemora and tibiae clearly incrassate, tarsi longest, slender, sphaerotrichomes missing (Figs 3A, B, 4B), claws simple, slightly curved (Fig. 4B, C); ♂ coxae 2 with short, membranous, cylindrical gonapophyses (Fig. 3G).

Gonopod aperture transversely oblong-oval, slightly subcordate, taking up most of ventral part of metazonite 7 (Fig. 4D). Gonopods (Fig. 4D–G) simple, with globose, scaly, medially fused coxae carrying a few setae on ventral face and a normal cannula mesally. Telopodites nearly straight, mostly exposed, *in situ* held parallel to each other, contiguous medially, largely unipartite due to prominent, rather densely setose prefemoral parts, rather short and stout, only distal quarter distinctly divided into a peculiar, axe-shaped, lateral solenophore (**sph**) and a smaller, anteriorly lying, sublanceolate solenomere (**sl**) directed slightly laterad. Seminal groove running entirely mesally, terminating on top of **sl.**

#### Aporodesmella similis sp. n.

http://zoobank.org/7DE6B4E3-4698-4D44-8B74-7E7B60BE07D0

http://species-id.net/wiki/Aporodesmella_similis

Figs 5 , 6

##### Type material.

Holotype ♂ (MNHN JC 356), Vietnam, Kien Giang Province, Ha Tien, Nui Da Dung, valley, 104.477E, 10.4288N, secondary forest, litter, Berlese extraction, 29.11.2006, leg. L. Deharveng & A. Bedos (Vn06-102).

Paratypes: 1 ♂ (SEM), same locality, together with holotype; 1 ♂, 1 ♀ fragment (MNHN JC 356), 1 ♀ (SEM), same locality, soil, Berlese extraction, 05.12.2005, leg. L. Deharveng & A. Bedos (Vn05-107); 1 ♂ (ZMUM ρ2347), same locality, litter, Berlese extraction, 05.12.2005, leg. L. Deharveng & A. Bedos (Vn05-112).

##### Name.

To emphasize the particularly strong similarity to the previous new species.

##### Diagnosis.

Differs from all congeners, except *Aporodesmella securiformis* sp. n., in the presence of a peculiar, dorsoparabasal stump on ♂ antennomere 6, from *Aporodesmella securiformis* sp. n. in the shape of the solenophore and solenomere.

##### Description.

Length of adults ca 3.0 mm, width of midbody pro- and metazonae 0.2 and 0.25 mm (♂). Coloration in alcohol uniformly pallid, tegument nearly translucent.

**Figure 5.**
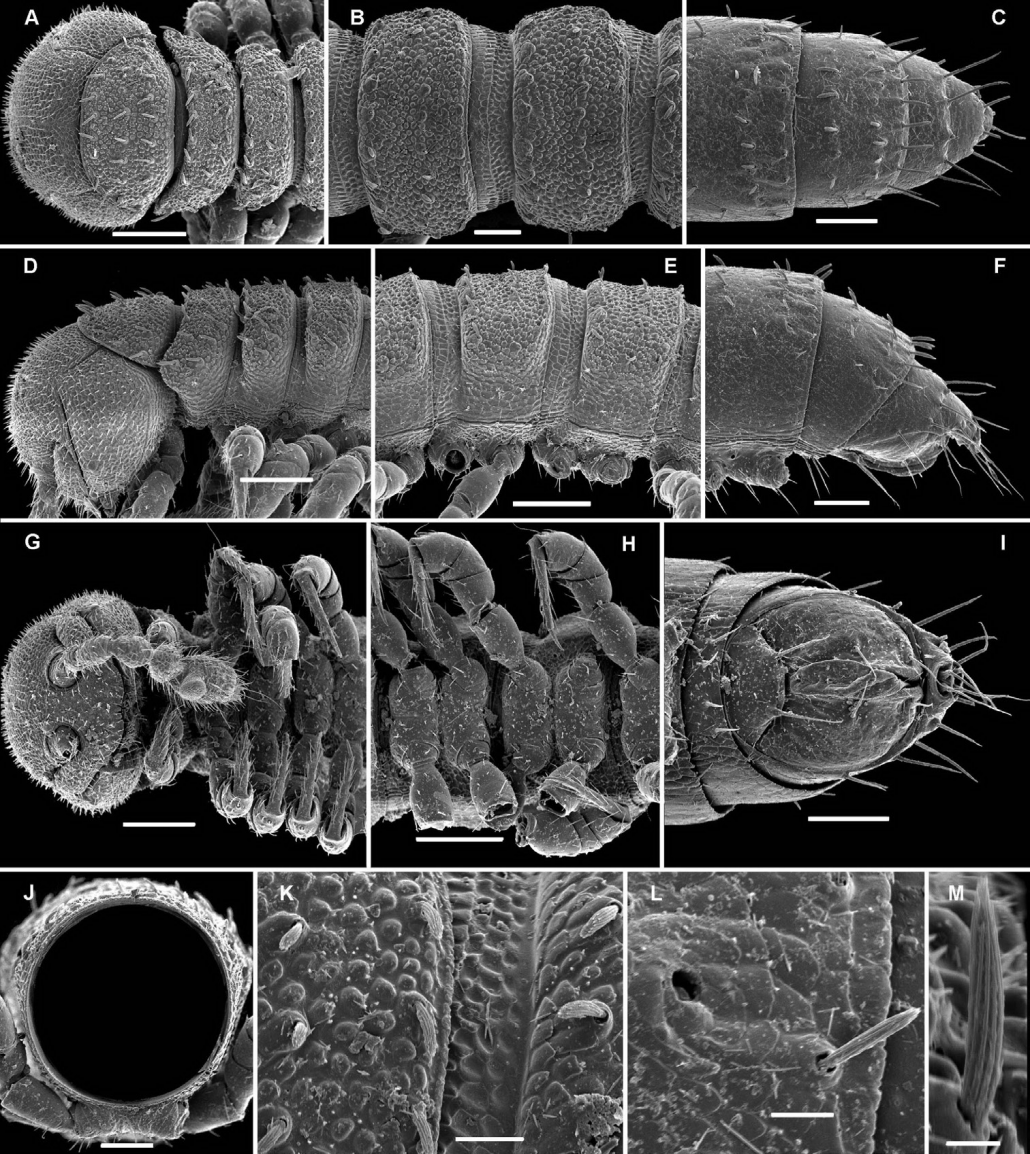
*Aporodesmella similis* sp. n., ♂ paratype; **A, D, G** anterior part of body, dorsal, lateral and ventral views, respectively **B, E, H** midbody segments, dorsal, lateral and ventral views, respectively **C, F, I** posterior part of body, dorsal, lateral and ventral views, respectively **J** cross-section of a midbody segment, caudal view **K, L** tergal setae, limbus and stricture region, subdorsal views **M** tergal seta, lateral view. Scale bars: A, **D, E, G, H** 0.1 mm; **B, C, F, I, J** 0.05 mm; **K** 0.02 mm; **L** 0.01 mm; **M** 0.005 mm.

Body moniliform, subcylindrical (Fig. 5A–J), with 19 segments (♂).

**Figure 6.**
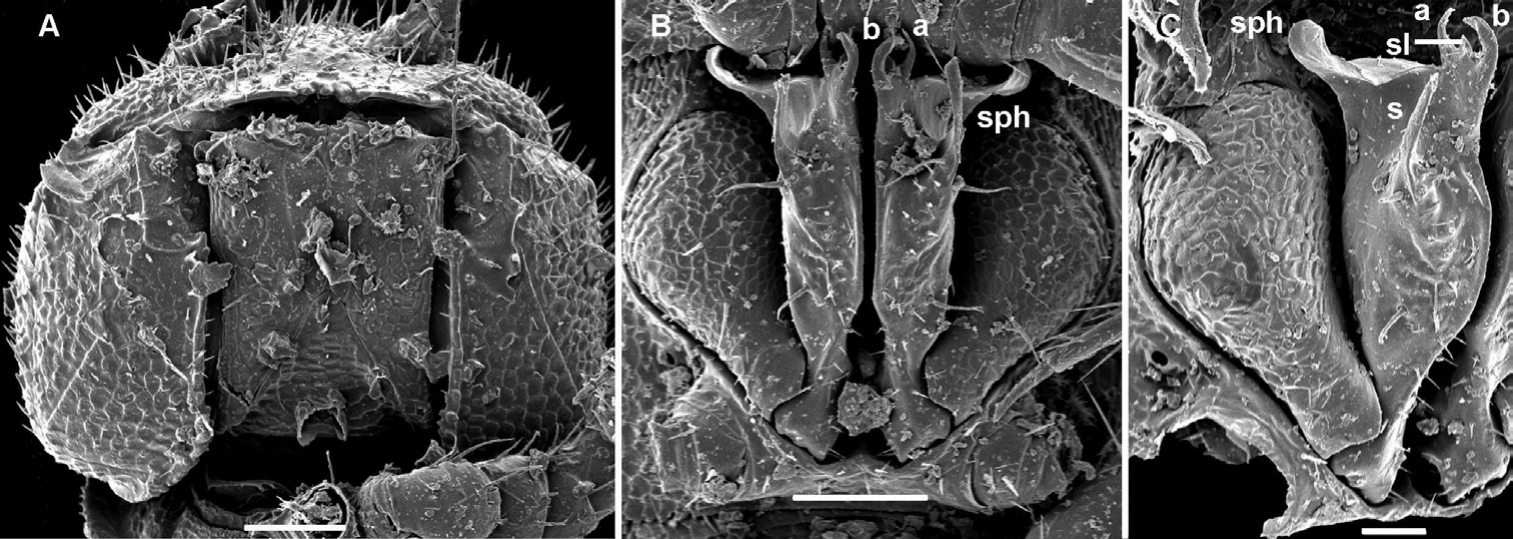
*Aporodesmella similis* sp. n., ♂ paratype; **A** head, ventral view **B** gonopod aperture and both gonopods *in situ*, ventral view **C** right gonopod, ventrolateral view. Scale bars: **A, B** 0.05 mm; **C** 0.02 mm. Designations of gonopod structures in text.

All characters as in *Aporodesmella securiformis* sp. n. (Figs 5, 6A), except as follows.

Gonopods (Fig. 6B, C) more complex, especially due to an elaborate and subtruncate tip of a more elongate and slenderer telopodite. Solenophore branch (**sph**) long, membranous, somewhat spoon-shaped, directed laterad, with a long, caudolateral, similarly membranous, subspiniform process (**s**) starting at base of **sph** and remaining coaxial with a prominent prefemoral portion. Mesal part of telopodite tip divided into 2 small horns (**a** and **b**) bearing a very small, dentiform solenomere (**sl**) lying in front and in between.

#### Aporodesmella tergalis sp. n.

http://zoobank.org/4B5EB78A-B952-4DB6-A5F1-F984EC2ADFFE

http://species-id.net/wiki/Aporodesmella_tergalis

Figs 7 , 8

Fuhrmannodesmidae gen. sp. – Golovatch et al. 2013: 73.

##### Type material.

Holotype ♂ (MNHN JC 357), Vietnam, Kien Giang Province, Kien Luong, Hon Chong, Nui Bai Voi, cirque du Français, 104.618799E, 10.218541N, litter, Berlese extraction, 23.08.2003, leg. L. Deharveng & A. Bedos (Vn0308-112).

Paratypes: 1 ♂, 1 ♀ (SEM), same locality, together with holotype.

##### Name.

To emphasize the well-developed paraterga.

##### Diagnosis.

Differs from congeners by well developed paraterga, short tergal setae, the ♂ also by the presence of a peculiar, central hump above the antennae, the laterally bent, beak-shaped solenophore and the absence of a solenomere.

##### Description.

Length of adults ca 4.0 mm, width of midbody pro- and metazonae 0.4 and 0.5 mm (♂). Coloration in alcohol uniformly pallid, tegument nearly translucent.

**Figure 7.**
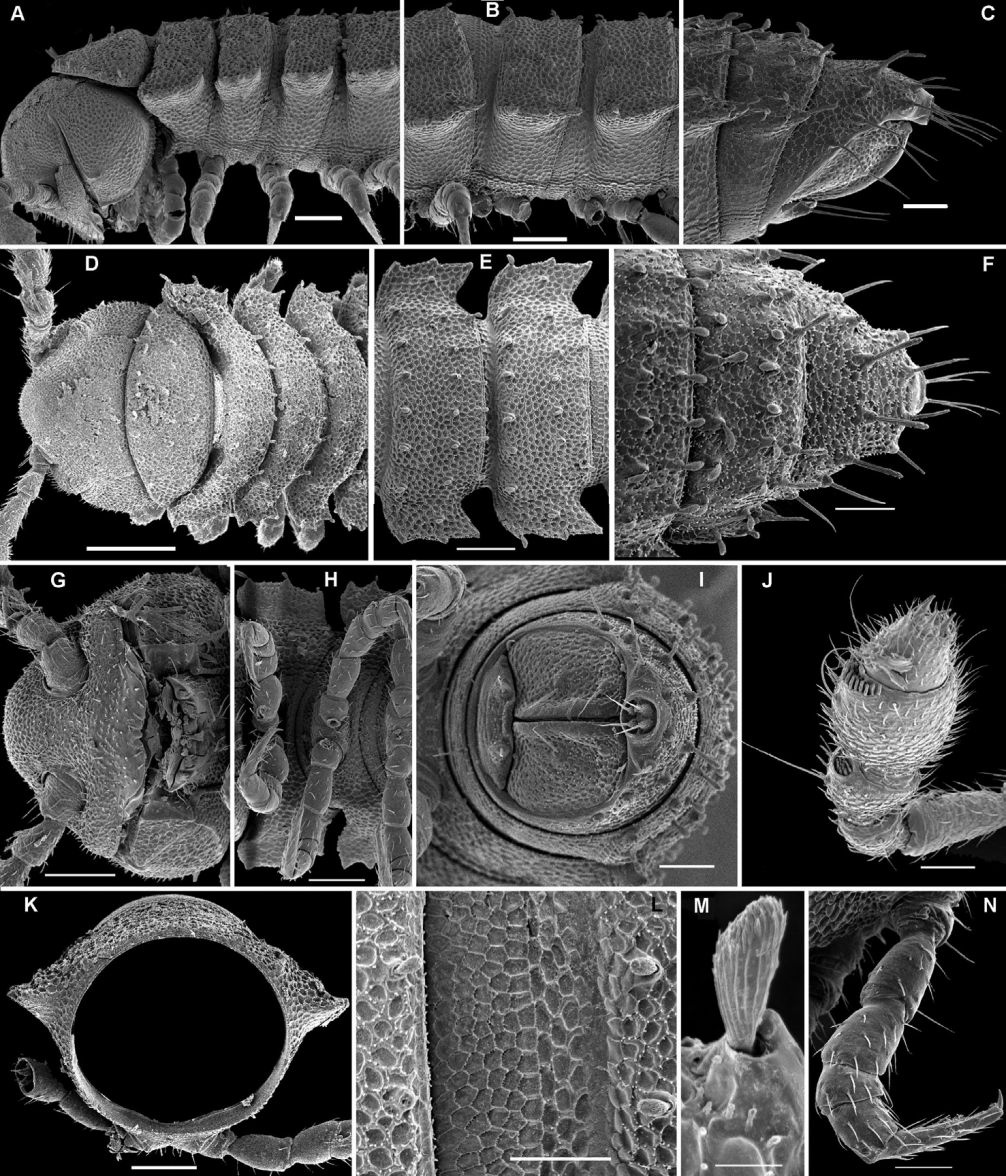
*Aporodesmella tergalis* sp. n., ♂ paratype; **A, D** anterior part of body, lateral and dorsal views, respectively **B, E** midbody segments, lateral and dorsal views, respectively **C, F, I** posterior part of body, lateral, dorsal and caudal views, respectively **G** head, ventral view **H** legs 1 and 2, ventral view **J** antenna, sublateral view **K** cross-section of a midbody segment, caudal view **L** tergal setae, limbus and stricture region, subdorsal view **M** tergal seta, lateral view **N** midbody leg. Scale bars: **D** 0.2 mm; **A, B, E, G, H, K** 0.1 mm; **C, F, I, J, L, N** 0.05 mm; **M** 0.005 mm.

**Figure 8.**
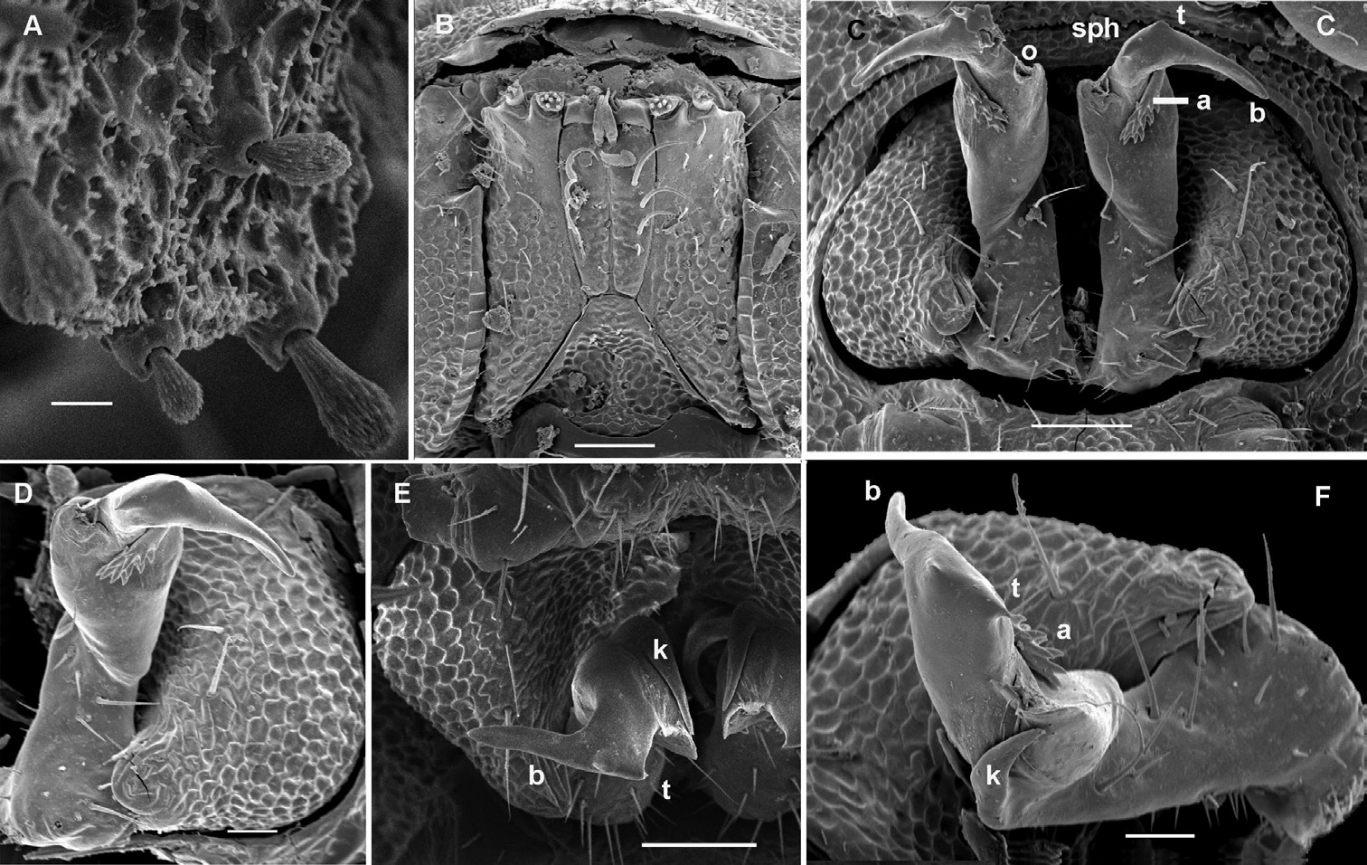
*Aporodesmella tergalis* sp. n., ♂ paratype; **A** cerategument and tergal setae, sublateral view **B** gnathochilarium, ventral view **C** gonopod aperture and both gonopods *in situ*, ventral view **D, F** left gonopod, subventral and submesal views, respectively **E** right gonopod, anteroventral view. Scale bars: **B, C, E** 0.05 mm; **D, F** 0.02 mm; **A** 0.01 mm. Designations of gonopod structures in text.

Body with 19 segments (♂). Tegument dull, texture delicately alveolate, a cerategument on metazonae well-developed (Fig. 8A). Head with an evident, round, very finely pilose, central hump above antennae (♂) (Fig. 7D, G), more roughly setose over remaining surface except occiput; epicranial suture superficial; genae squarish (Fig. 7A, D, G); gnathochilarium narrow, setae on lamellae linguales particularly strong (Fig. 8B); isthmus between antennae about 1.5 times as broad as diameter of antennal socket (Fig. 7G). Antennae very short, reaching only behind collum when stretched dorsally, not geniculate, strongly clavate due to an abruptly and particularly enlarged antennomere 6, the latter with a usual, tight, distodorsal group of numerous bacilliform sensilla, antennomere 5 with a smaller, but also compact, distodorsal group of only a few shorter sensilla, 7^th^ with a tiny mid-dorsal knob (Fig. 7J).

In width, head = collum < segment 3 = 4 < 2 < 5 = 16 (♂), thereafter body gradually tapering towards telson (Fig. 7D–F). Paraterga well-developed, set low (at about upper 1/3-1/2 of body height), mostly clearly declined (Fig. 7A–F, K), front edges moderately convex and forming a shoulder, caudal edges increasingly strongly concave caudad, lateral edges mostly straight, on each side with 3 setigerous, equidistant knobs; caudal corners mostly nearly sharp, lying within rear tergal margin until segment 10, thereafter increasingly well produced behind the margin (Fig. 7A–F). Ozopores totally absent (Figs 7A–F, I, 8A). Collum biconvex, nearly sharp laterally on both sides, with 3 transverse rows of short setae (Fig. 7D). Each following metatergum mostly with 3+3 short, slightly blunted, clavate, thickened and longitudinally ribbed setae arranged in 3 transverse regular rows and borne on minute knobs; sulci between the rows absent (Figs 7A–F, M, 8A). Stricture between pro- and metazonae rather shallow and narrow, scaly like rear part of prozonae (Fig. 7E, L). Limbus very fine, very delicately and sparsely microdenticulate (Fig. 7L). Pleurosternal carinae absent (Fig. 7A, B, H). Epiproct short, conical, truncate, directed caudoventrally; pre-apical papillae large (Fig. 7C, F, I). Hypoproct subtrapeziform, caudal setigerous papillae well-developed and clearly separated (Fig. 7I).

Sterna without modifications, rather broad and sparsely setose. Legs short, ca 1.2–1.3 times as long as midbody height (♂); prefemora, femora, postfemora and tibiae clearly incrassate, tarsi longest, slender, sphaerotrichomes missing; claws simple, slightly curved (Fig. 7N); ♂ coxae 2 with very short, membranous, cylindrical gonapophyses (Fig. 7H).

Gonopod aperture transversely oblong-oval, slightly subcordate, taking up most of ventral part of metazonite 7 (Fig. 8C). Gonopods (Fig. 8C–F) rather complex, with globose, microgranulate, medially fused coxae carrying a few setae on ventral face and a normal cannula mesally. Telopodites mostly exposed, *in situ* held parallel to each other, nearly contiguous medially, each unipartite, with a rather large, densely setose prefemoral part clearly set off from acropodite by an oblique ventral sulcus and a curved dorsal spine (**k**); acropodite divided into 2 subequally long parts, each also about equal in length to prefemoral portion; basal half of acropodite remaining coaxial with prefemoral portion, slightly broadened distad and carrying an evident orifice (**o**) of a fully mesally running seminal groove on a vestigial mesal solenomere (= tubercle), whereas distal half of acropodite, or solenophore (**sph**), acuminate, directed abruptly laterad, subflagelliform (**b**), carrying a parabasal apical tooth (**t**) and a conspicuous, curved, denticulate lamina (**a**) beginning from near **o** and turning around **b** on lateral side.

##### Remarks.

This species served elsewhere (Golovatch et al. 2013) to illustrate a somewhat transitional condition in its gonopod structure, especially an elongate and laterad directed apical part, between the Trichopolydesmidae (= “Fuhrmannodesmidae”) and Opisotretidae within the same superfamily Trichopolydesmoidea. A far more spectacular example of the same trend is seen in *Gonatodesmus* gen. n. (see below).

#### Deharvengius gen. n.

http://zoobank.org/063BDEC3-966B-4AC3-98CF-CE76FDF85D86

http://species-id.net/wiki/Deharvengius

##### Diagnosis.

18 segments (♂, ♀); pore formula normal: 5, 7, 9, 10, 12, 13, 15–17; head modified in both sexes in being somewhat flattened dorsoventrally; paraterga very poorly developed, serrate/microdentate at lateral edge, with 3 rows of 2+2, long, simple setae (regardless of lateral setae); gonopod coxae with gonocoel not deep; telopodites clearly exposed, but lying tightly appressed and parallel to venter, strongly curved, semi-circular, unipartite, slender, directed mesad and strongly overlapping; prefemoral parts about half as long as telopodites, set off from acropodites neither by a sulcus nor a cingulum; acropodites with small solenophores (**sph**) lying basal to a spiniform, apical solenomere (**sl**). Seminal groove running mostly along ventral surface of subbasally obviously twisted acropodites.

##### Name.

To honour Louis Deharveng (MNHN), one of the principal collectors, masculine.

##### Type species.

*Deharvengius bedosae* sp. n., by present designation.

##### Remarks.

In having only 18 body segments, this new genus is almost unique amongst the Trichopolydesmoidea. The same segment counts seem to solely concern *Moojenodesmus pumilus* Schubart, 1944, only one of the species of the rather small Neotropical genus *Moojenodesmus* Schubart, 1945, from the same family Trichopolydesmidae; *Moojenodesmus pumilus* is especially minute (< 2.5 mm long), and it seems to be parthenogenetic and quite widespread in Brazil (Golovatch 1992, 1994). Among the Oriental Trichopolydesmidae, partly globally as well, *Deharvengius* gen. n. stands well apart also in having a less convex head, only 2+2 tergal setae per row and very unusual gonopods. The latter are long, simple, unipartite, strongly curved and crossing medially, thus being quite similar to the condition observed in *Cocacolaria* (Fig. 2B–E). Yet in *Deharvengius* gen. n. the gonopods are much more strongly appressed to the venter while the seminal groove runs mostly on the ventral = lateral (not mesal) side to terminate apically on a simple and slender (not at about midway on a stout and calyciform) solenomere. By its habitus and even gonopod structure, *Deharvengius* gen. n. resembles some Euro-Mediterranean genera of Trichopolydesmidae as well (see reviews by Mauriès (1983) and Golovatch et al. (2013)). Some of them show a deeply bipartite and strongly curved gonopod telopodite, the prefemoral part of which is quite elongate, but lies more or less transversely to strongly angular, largely (sub)parallel telopodites and extends across the nearly entire ventral width of segment 7. Such are *Trichopolydesmus* Verhoeff, 1898 (together with the subgenus *Banatodesmus* Tabacaru, 1980), *Bacillidesmus* Attems, 1898, *Napocodesmus* Ceuca, 1974 and *Caucasodesmus* Golovatch, 1985. In contrast, the gonotelopodites in *Verhoeffodesmus* Strasser, 1959, *Cottodesmus* Verhoeff, 1936 and *Occitanocookia* Mauriès, 1980 have increasingly shortened prefemoral parts, being enlarged and laterally flattened distad and unipartite, but mostly less strongly curved, in *Cottodesmus* and *Occitanocookia* also devoid of a solenomere, but sometimes supplied instead with what can be seen as a primordial accessory seminal chamber. In *Trichopolydesmus*, *Heterocookia* Silvestri, 1898, *Ingurtidorgius* Strasser, 1974 and, especially, *Mastigonodesmus* Silvestri, 1898, the solenomere is almost to fully flagelliform, branching off near the base of the femorite. In all these genera, the gonotelopodites are strongly exposed, not sunken inside an obvious gonocoel (Golovatch et al. 2013).

*Deharvengius* gen. n. currently contains only one species.

#### Deharvengius bedosae sp. n.

http://zoobank.org/5C60DC06-021E-402F-8D03-871532155442

http://species-id.net/wiki/Deharvengius_bedosae

Figs 9 , 10

##### Type material.

Holotype ♂ (MNHN JC 358), Vietnam, Kien Giang Province, Kien Luong, Hon Chong, Nui Bai Voi (cirque sud), 104.617E, 10.2199N, soil, Berlese extraction, 26.11.2008, leg. L. Deharveng & A. Bedos (Vn06-30).

Paratypes: 2 ♀, 4 juv. (subadults) (MNHN JC 358), same province, Kien Luong, Hon Chong, Nui Bai Voi (cirque sud), 104.617E, 10.2199N; soil, Berlese extraction, 02.06.2008, leg. L. Deharveng & A. Bedos (Vn08-055); 1 ♂, 2 ♀ (MNHN JC 358), 1 ♂ (ZMUM ρ2348), 1 ♂, 1 juv. (subadult) (SEM), same province, Hon Chong, Nui Khoe La, soil, Berlese extraction, 01.06.2008, leg. L. Deharveng & A. Bedos (Vn08-027).

##### Name.

To honour Anne Bedos (MNHN), one of the principal collectors.

##### Description.

Length of adults ca 3.0 mm, width of midbody pro- and metazonae 0.3 and 0.45 mm (♂, ♀). Coloration in alcohol uniformly pallid, tegument often nearly translucent.

**Figure 9.**
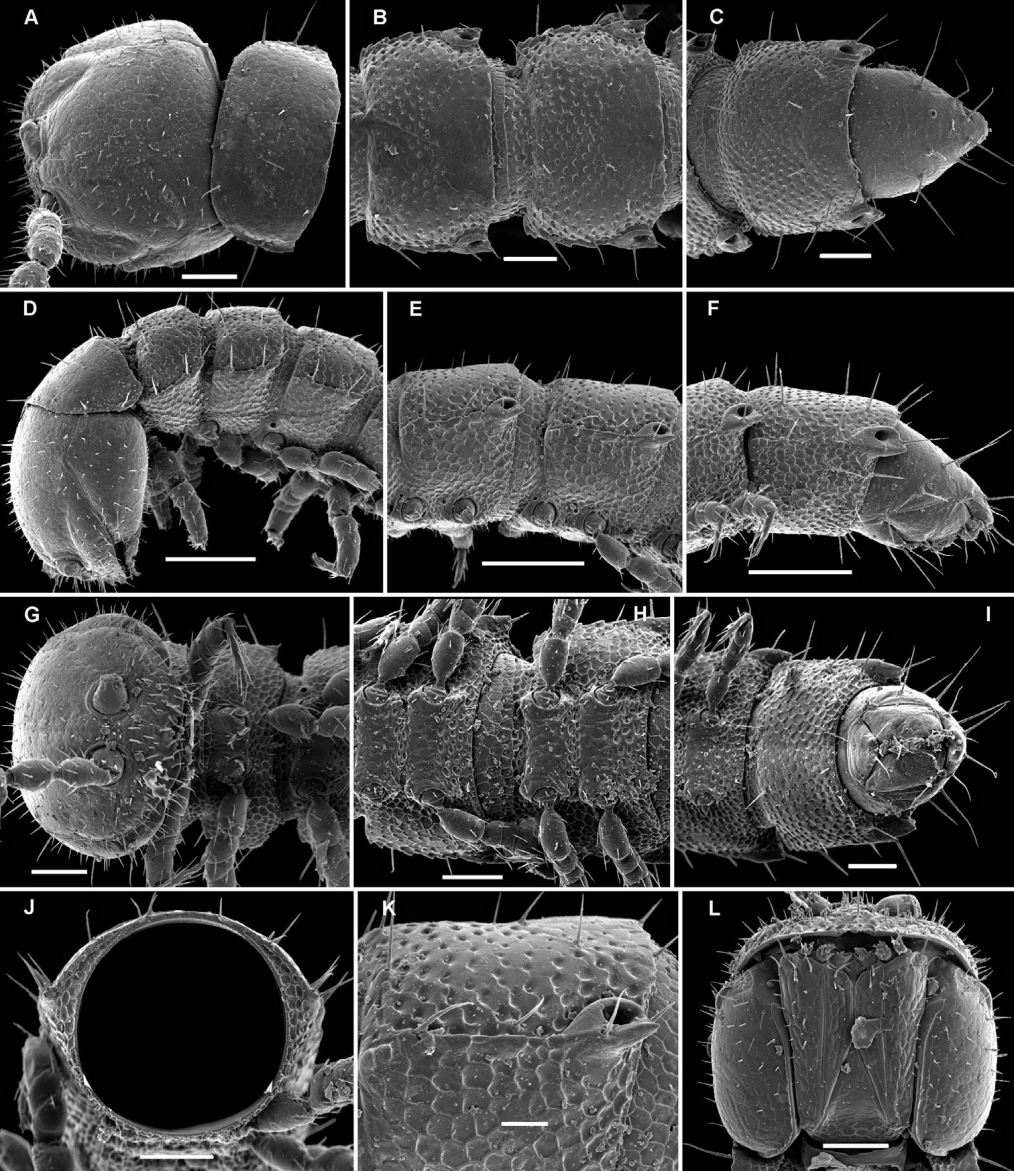
*Deharvengius bedosae* sp. n., ♂ paratype from Nui Khoe La; **A, D, G** anterior part of body, dorsal, lateral and ventral views, respectively **B, E, H** midbody segments, dorsal, lateral and ventral views, respectively **C, F, I** posterior part of body, dorsal, lateral and ventral views, respectively **J** cross-section of a midbody segment, caudal view **K** poriferous midbody paratergite, tergal setae, tegument structure, limbus and stricture region **L** head, ventral view. Scale bars: **D–F** 0.1 mm; **A–C, G–J, L** 0.05 mm; **K** 0.02 mm.

**Figure 10.**
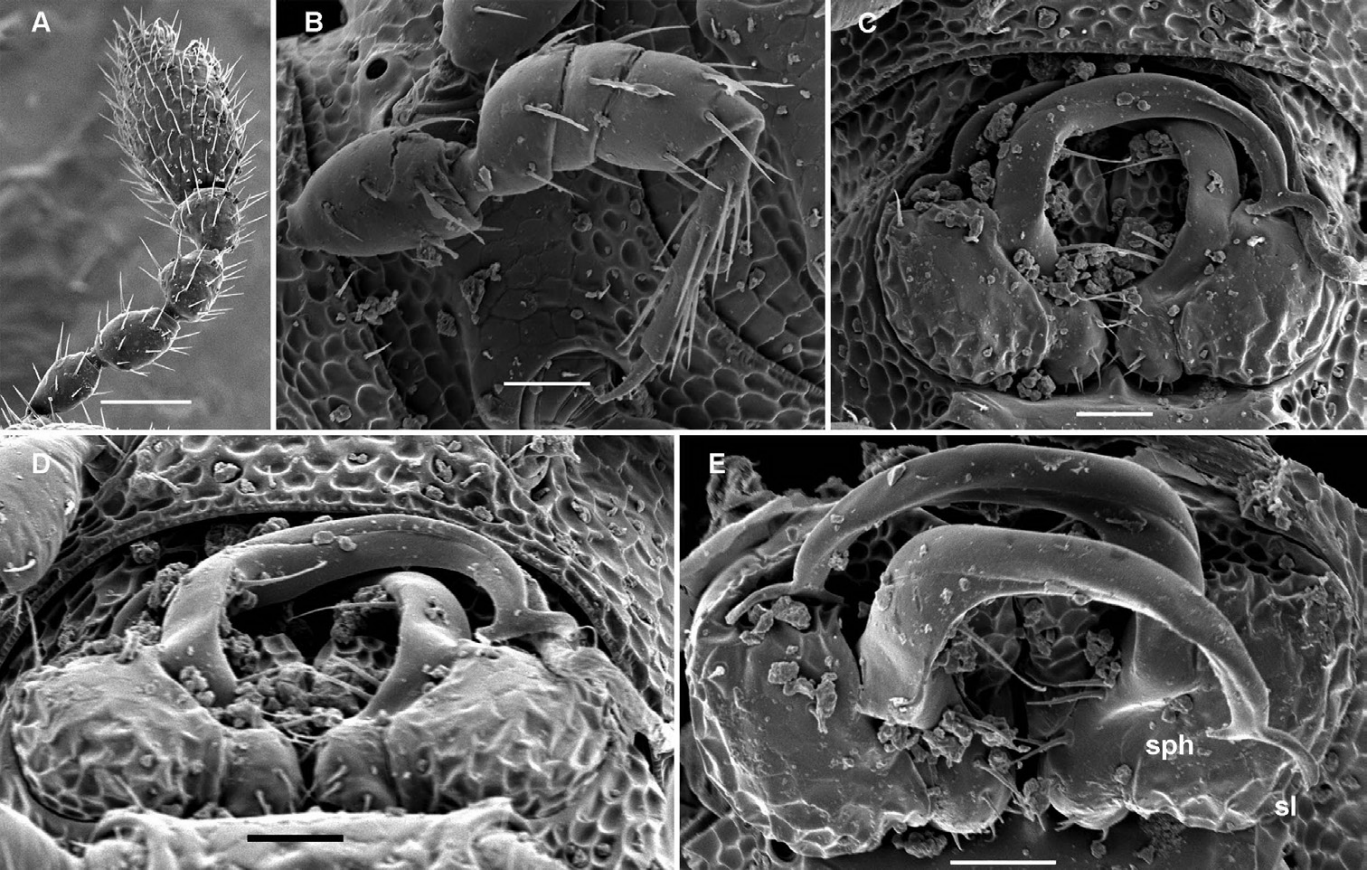
*Deharvengius bedosae* sp. n., ♂ paratype from Nui Khoe La; **A** antenna, lateral view **B** midbody leg, lateral view **C–E** gonopod aperture and both gonopods *in situ*, ventral, ventrocaudal and anteroventral views, respectively. Scale bars: **A** 0.05 mm; **B–E** 0.02 mm Designations of gonopod structures in text.

Body with 18 segments (♂, ♀). Tegument dull, texture of metazonae very delicately punctate on dorsum and sterna, but alveolate laterally below paraterga; collum smooth (Fig. 9A–K). Head relatively sparsely and finely pilose, less convex than usual (♂, ♀); epicranial suture superficial; genae squarish (Fig. 9A, D, G, L); gnathochilarium narrow, sparsely and uniformly setose (Fig. 9L); isthmus between antennae about 0.8 times as broad as diameter of antennal socket (Fig. 9A, G). Antennae very short (Fig. 10A), reaching only behind head when stretched dorsally, not geniculate, strongly clavate due to an abruptly and particularly enlarged antennomere 6, the latter with a usual, tight, distodorsal group of rather numerous, bacilliform sensilla; antennomere 7 with a smaller distodorsal group of only a few shorter and curved sensilla in front of a tiny mid-dorsal knob.

Body moniliform, subcylindrical (Fig. 9A–J). In width, head > segments 5-15 > 2 > 3 = 4 > collum; body gradually tapering on segments 16-18 (Fig. 9A–I). Paraterga very poorly developed, starting from collum, set low (at about upper 1/3-1/2 of body height), mostly represented by vestigial, delicately serrate ridges and sharp caudal teeth, the latter being clearly enlarged and slightly produced behind rear tergal margin in poriferous segments (Fig. 9A–K). Ozopores evident, ovoid, dorsolateral, lying about equally close to caudal corner and lateral edge (Fig. 9B, C, E, F, K). Collum roundly subquadrate, with 3 transverse rows of long setae dorsally and 2 similar setae on paraterga (Fig. 9A). Each following metatergum mostly with 2+2 long and pointed setae arranged in 3 transverse regular rows and not borne on knobs; sulci between the rows absent (Fig. 9B–F). Stricture between pro- and metazonae rather deep and narrow, scaly like rear part of prozonae (Fig. 9B, E). Limbus very fine, very delicately and sparsely microdenticulate (Figs 9J, K, 10D). Pleurosternal carinae absent (Fig. 9D–F). Epiproct short, conical, truncate, directed caudoventrally; pre-apical papillae small (Fig. 9C, F, I). Hypoproct subtrapeziform, relatively high and narrow, caudal setigerous papillae large and moderately separated, with a faintly convex edge in between (Fig. 9I).

Sterna without modifications, rather broad and sparsely setose (Fig. 9G–I). Legs short, ca 1.2–1.3 (♂) or 1.0–1.1 (♀) times as long as midbody height; prefemora, femora, postfemora and tibiae clearly incrassate, especially so in ♂ (Fig. 10B), tarsi longest, slender, sphaerotrichomes missing; claws simple, slightly curved; ♂ coxae 2 with very short, membranous, cylindrical gonapophyses (Fig. 9G).

Gonopod aperture transversely oblong-oval, taking up most of ventral part of metazonite 7 (Fig. 10C–E). Gonopod coxae with gonocoel not deep; telopodites clearly exposed, but lying tightly appressed and parallel to venter, strongly curved, semi-circular, unipartite, slender, directed mesad and strongly overlapping; prefemoral parts about half as long as telopodites, set off from acropodites neither by a sulcus nor a cingulum; each acropodite with a small solenophore (**sph**) lying basal to a spiniform, apical solenomere (**sl**). Seminal groove running mostly along ventral (= lateral) surface of a subbasally obviously twisted acropodite.

#### Gonatodesmus gen. n.

http://zoobank.org/7F9F0843-BD27-4A71-8995-91722D9CB039

http://species-id.net/wiki/Gonatodesmus

##### Diagnosis.

19 segments (♂, ♀); pore formula normal: 5, 7, 9, 10, 12, 13, 15-18; ♂ head with a considerable, rounded, central hump above antennae; paraterga poorly developed, serrate/microdentate at lateral edge, with 3 rows of 3+3 or 4+4, long, bacilliform setae (regardless of lateral setae); gonopod coxae with gonocoel not deep; telopodites clearly exposed, but lying rather tightly appressed and mostly parallel to venter, unipartite, slender, abruptly geniculate and directed laterad distal to prefemoral parts, the latter about half as long as telopodites, held parallel to main axis, each set off from a twisted acropodite by a geniculation cingulum (**c**); acropodites elongate, near midway with a remarkably large hairy pulvillus (**p**) on top of a small accessory seminal chamber; neither a separate solenophore branch nor a solenomere. Seminal groove (**sg**) running mostly on mesal side, turning caudad (= ventrad) only beyond geniculation.

##### Name.

To emphasize the geniculated gonopod telopodite (γόνατο meaning “a knee” in Greek), masculine.

##### Type species.

*Gonatodesmus communicans* sp. n., by present designation.

##### Remarks.

This new genus is highly disjunct in being the sole member of the family which shows a remarkable midway geniculation of the gonopod telopodite. The gonopod tip being directed laterad is not unique in Trichopolydesmidae, shared also with *Aporodesmella tergalis* sp. n. However, the entire distal, post-geniculation half of the gonopod in *Gonatodesmus* gen. n. is obviously twisted and strongly elongate, also showing a remarkably distinct hairy pulvillus on top of a small, but evident accessory seminal chamber. Only vestiges of the latter are occasionally observed in a couple of Euro-Mediterranean genera of Trichopolydesmidae (Golovatch 2013), whereas an obvious hairy pulvillus seems to be singular in the family. In contrast, such a gonopod conformation vividly resembles the conditions largely characterizing the family Opisotretidae (Golovatch et al. 2013), but the prefemoral parts in *Gonatodesmus* gen. n. are clearly elongate and held parallel to the main axis while the acropodites distal to the unique geniculation are yet relatively short to be directed dorsolaterad. So the assignment of *Gonatodesmus* gen. n. to Trichopolydesmidae, not Opisotretidae, is preferred. However, one may justly regard *Gonatodesmus* gen. n. as a kind of transition or bridge between these two families of Trichopolydesmoidea. This new genus rather demonstrates an evolutionary trend from the more basal Trichopolydesmidae towards the advanced Opisotretidae.

#### Gonatodesmus communicans sp. n.

http://zoobank.org/3B7B82CD-75D0-4444-9E99-4FB6C7741F60

http://species-id.net/wiki/Gonatodesmus_communicans

Figs 11 , 12

##### Type material.

Holotype ♂ (MNHN JC 359), Vietnam, Dongnai Prov., Cat Tien National Park, 107°10'–107°34'E, 11°21'–11°48'N, lowland tropical forest, litter and topsoil, 01.06.2005, leg. A. Anichkin.

Paratypes: 5 ♂, 1 ♀ (MNHN JC 359), 9 ♂, 1 ♀, 1 juv. (ZMUM ρ2336–2339), 1 ♂ (SEM), 1 ♂ (ZMUC), same locality, together with holotype; 1 ♀ (MNHN JC 359), same locality, 15.07.2005, leg. A. Anichkin.

##### Name.

The species epithet refers to the gonopod conformation somewhat transitional between the families Trichopolydesmidae and Opisotretidae.

##### Description.

Length of adults ca 2.8-3.7 mm, width of midbody pro- and metazonae 0.2–0.25 and 0.28–0.4 mm (♂, ♀). Coloration in alcohol uniformly pallid, tegument often nearly translucent.

**Figure 11.**
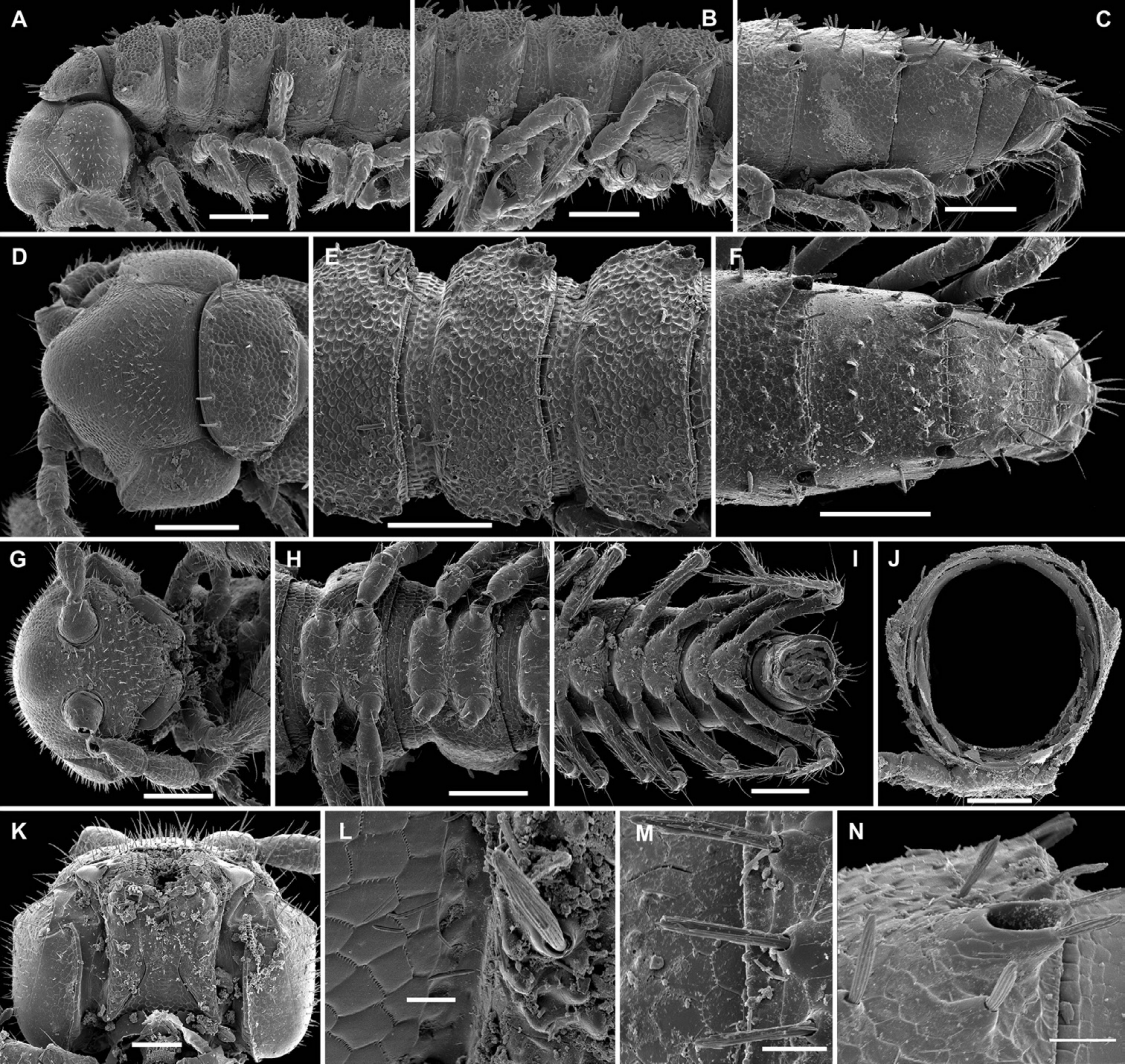
*Gonatodesmus communicans* sp. n., ♂ paratype; **A, D, G** anterior part of body, lateral, dorsal and ventral views, respectively **B, E, H** midbody segments, lateral, dorsal and ventral views, respectively **C, F, I** posterior part of body, lateral, dorsal and ventral views, respectively **J** cross-section of a midbody segment, caudal view **K** head, ventral view **L, M** poriferous midbody paratergite, tergal setae, tegument structure, limbus and stricture region **N** ozopore region of segment 16, subdorsal view. Scale bars: **A–D, F–J** 0.1 mm; **E, K** 0.05 mm; **N** 0.02 mm; **L, M** 0.01 mm.

**Figure 12.**
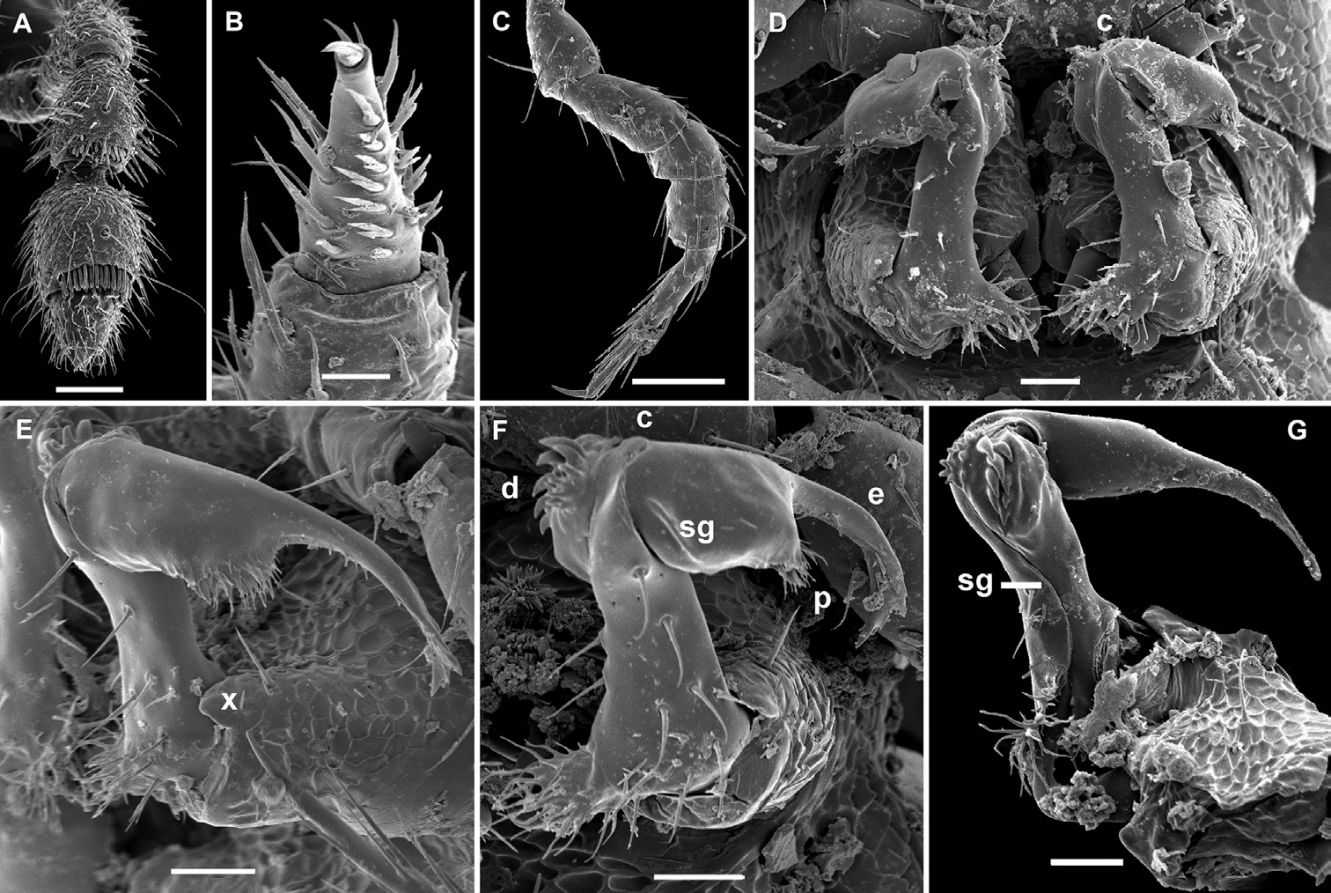
*Gonatodesmus communicans* sp. n., ♂ paratype; **A** antennomeres 4-8, dorsal view **B** tarsus 1, ventral view **C** midbody leg, lateral view **D** both gonopods *in situ*, ventral view **E–G** left gonopod, anteroventral, ventral and submesal views, respectively. Scale bars: **A, C** 0.05 mm; **D–G** 0.02 mm; **B** 0.01 mm. Designations of gonopod structures in text.

Body with 19 segments (♂, ♀). Tegument dull, texture of pro- and metazonae delicately alveolate to scaly, metaterga a little more roughly alveolate, only sterna mostly smooth (Fig. 11A–I). Head very densely and finely pilose; epicranial suture absent (♂) or superficial (♀); genae squarish (Fig. 11A, D, G, K); gnathochilarium narrow, sparsely and uniformly setose (Fig. 11K); isthmus between antennae about 1.2 times as broad as diameter of antennal socket (Fig. 11G). Antennae short (Figs 11A, G, 12A), reaching only behind collum when stretched dorsally, not geniculate, rather strongly clavate due to a particularly enlarged antennomere 6, the latter with a usual, tight, distodorsal group of numerous, bacilliform sensilla; a similar, but smaller, also distodorsal group of sensilla on antennomere 5; antennomere 7 with a smaller distodorsal group of only a few shorter and curved sensilla in front of a tiny mid-dorsal knob.

In width, collum < segment 3 = 4 < 2 = 5 < 6–15 < head; body gradually tapering on segments 16–18 (Fig. 11A–I). Paraterga poorly developed, starting from collum, set rather low (at about upper 1/3 of body height, Fig. 11J), at most small ridges with 2–3 lateral, setigerous knobs, caudal corner never produced behind rear tergal margin even in poriferous segments (Fig. 11A–F, N). Ozopores evident, ovoid, dorsolateral, mostly lying closer to lateral edge, but in segments 15-18 slightly elevated and positioned closer to caudal edge between 2 subequal, setigerous stalks (Fig. 11N). Collum regularly rounded, transversely oblong-oval, with 3 transverse rows of 4+4, 2+2 and 3+3 rather long setae dorsally and 2 similar setae on paraterga (Fig. 11A, D). Each following metatergum with 3+3 or 4+4, similarly long, bacilliform, delicately ribbed setae arranged in 3 transverse regular rows and borne on knobs; sulci between the rows absent (Fig. 11A–F, L–N). Stricture between pro- and metazonae rather deep and narrow, microalveolate like metazonae (Fig. 11E, L, N). Limbus very fine, delicately and densely microcrenulate (Fig. 11E, N). Pleurosternal carinae absent (Fig. 11A–C). Epiproct short, conical, truncate, directed caudoventrally; pre-apical papillae very small (Fig. 11C, F). Hypoproct subtrapeziform, caudal setigerous papillae large and well separated, with a faintly convex edge in between (Fig. 11I).

Sterna without modifications, rather broad and sparsely setose (Fig. 11H, I). Legs short and stout, ca 1.2–1.3 (♂) or 1.0–1.1 (♀) times as long as midbody height; prefemora, femora, postfemora and tibiae clearly incrassate, especially so in ♂ (Fig. 12C), tarsi longest, slender, sphaerotrichomes missing; claws simple, slightly curved; ♂ tarsi 1 with peculiar, bi- or trifid setae ventrally (Fig. 12B); ♂ coxae 2 with very short, membranous, cylindrical gonapophyses (Fig. 11G).

Gonopod aperture transversely oblong-oval, taking up most of ventral part of metazonite 7 (Fig. 12D). Gonopod coxae (Fig. 12D–G) rather small (gonocoel not deep), sparsely setose and clearly micropapillate laterally, each with a subtriangular ventral projection (**x**); telopodites clearly exposed, but lying rather tightly appressed and parallel to venter, unipartite, slender, abruptly geniculate and directed laterad distal to prefemoral parts, the latter about half as long as telopodites, held parallel to main axis, each with an apicomedian field of strong curved spines (**d**), set off from a twisted acropodite by a strong geniculation cingulum (**c**); acropodite elongate, near midway with a remarkably large hairy pulvillus (**p**) on top of a small accessory seminal chamber; acropodite distal to pulvillus particularly slender, slightly curved caudad, subacuminate; neither a separate solenophore branch nor a solenomere. Seminal groove (**sg**) running mostly on mesal side, turning caudad (= ventrad) only beyond geniculation.

#### Helicodesmus gen. n.

http://zoobank.org/77E87B7A-EABE-494E-A6C1-4BB38BA7E280

http://species-id.net/wiki/Helicodesmus

##### Diagnosis.

19 segments (♂, ♀); pore formula normal: 5, 7, 9, 10, 12, 13, 15–18; head without modifications; paraterga poorly developed, metaterga with 3 rows of 3+3 or, more rarely, 4+4, long, bacilliform setae (regardless of lateral setae); gonopod coxae with gonocoel not deep; telopodites rather clearly exposed and transverse, but stout and remarkably complex, very strongly twisted, partly fimbriate/plumose distally, with several outgrowths of varying shapes; prefemoral part about half the height of telopodite, demarcated on lateral (not medial!) side by an oblique seminal groove running further mesad onto a medioventral outgrowth of acropodite to terminate distally, with neither a solenomere nor an accessory seminal chamber, nor a pulvillus.

##### Name.

To emphasize the strongly helicoid gonopod telopodite, masculine.

##### Type species.

*Helicodesmus anichkini* sp. n., by present designation.

##### Remarks.

This new genus is remarkable within Trichopolydesmidae in showing particularly strongly twisted gonopods, including their prefemoral parts, such that the seminal groove turns ca 180° around the highly complex transverse acropodite. Also noteworthy is the complete lack of a solenomere.

#### Helicodesmus anichkini sp. n.

http://zoobank.org/981D48EE-2D59-4940-8954-4CA702DD2B5C

http://species-id.net/wiki/Helicodesmus_anichkini

Figs 13 – 15

##### Type material.

Holotype ♂ (MNHN JC 360), Vietnam, Dongnai Prov., Cat Tien National Park, 107°10'–107°34'E, 11°21'–11°48'N, lowland tropical forest, litter and topsoil, 01.06.2005, leg. A. Anichkin.

Paratypes: 2 ♂, 1 ♀ (ZMUM ρ2340, ρ2343), 1 ♂ (SEM), same locality, together with holotype; 1 ♂ (ZMUM ρ2342), same locality, 01.04.2005; 2 ♂ (MNHN JC 360), 1 ♂ (ZMUM ρ2341), same locality, 15.07.2005; 1 ♂, 1 ♀ (MNHN JC 360), 2 ♂ (ZMUM ρ2344, ρ2345), same locality, 23.11.2005; 1 ♂ (MNHN JC 360), same locality, 17.07.2005; 1 ♂ (ZMUC), same locality, 15.05.2005, all leg. A. Anichkin.

##### Name.

To honour Alexander E. Anichkin, who provided for study all millipede material he had collected in Cat Tien National Park, including three trichopolydesmids described here.

##### Description.

Length of adults ca 3.0-4.0 mm, width of midbody pro- and metazonae 0.28-0.32 and 0.4-0.45 mm (♂, ♀). Coloration in alcohol uniformly pallid, tegument often nearly translucent.

**Figure 13.**
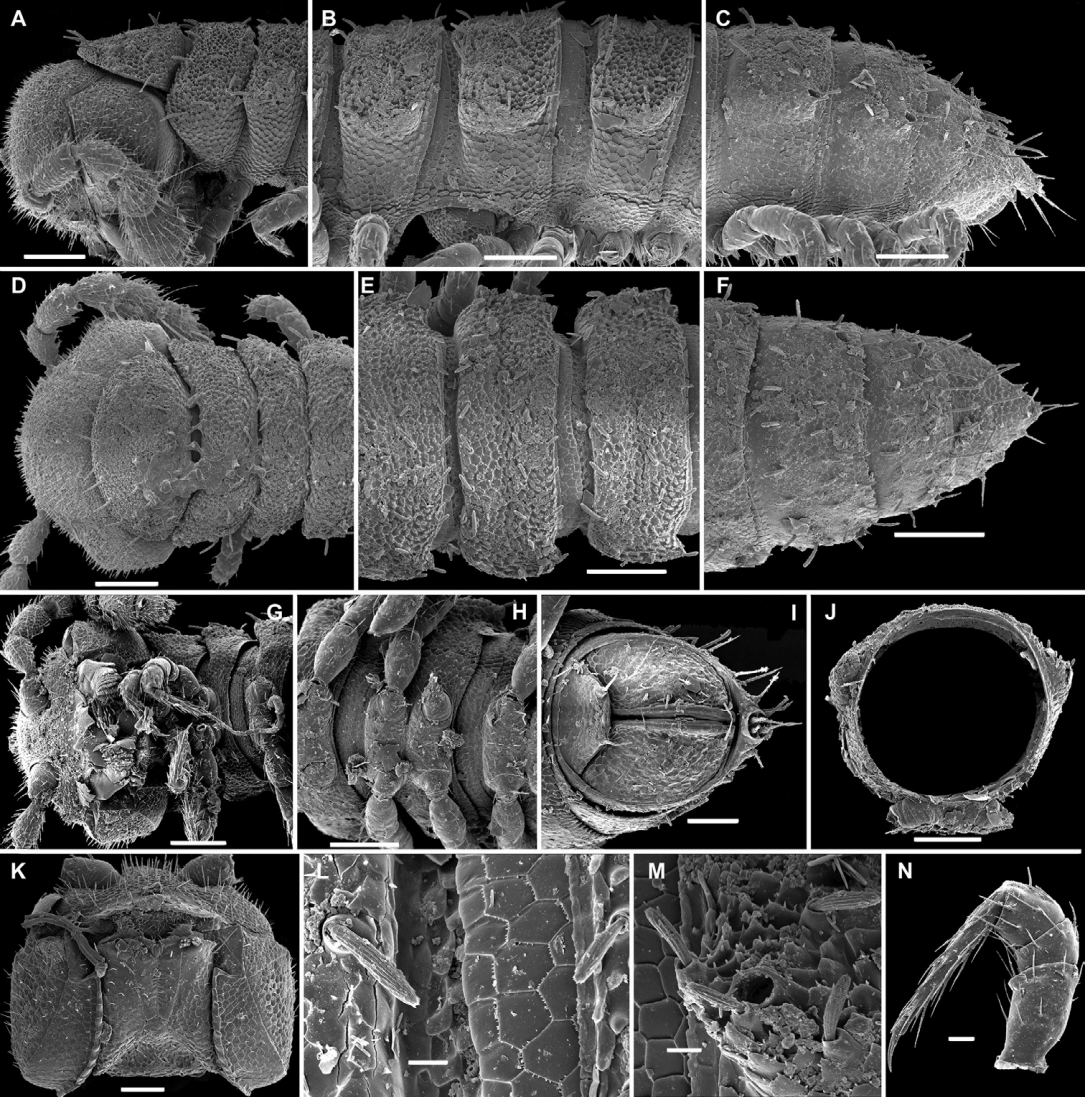
*Helicodesmus anichkini* sp. n., ♂ paratype; **A, D, G** anterior part of body, lateral, dorsal and ventral views, respectively **B, E, H** midbody segments, lateral, dorsal and ventral views, respectively **C, F, I** posterior part of body, lateral, dorsal and ventral views, respectively **J** cross-section of a midbody segment, caudal view **K** head, ventral view **L** tergal setae, tegument structure, limbus and stricture region **M** ozopore region of a midbody segment, sublateral view **N** midbody leg, lateral view. Scale bars: **A–J** 0.1 mm; **K** 0.05 mm; **N** 0.02 mm; **L, M** 0.01 mm.

Body with 19 segments (♂, ♀). Tegument dull, texture of nearly entire body delicately alveolate to scaly, only sterna nearly smooth (Fig. 13A–I). Head very densely and finely pilose; epicranial suture highly superficial; genae squarish (Fig. 13A, D, G, K); gnathochilarium rather broad, sparsely and uniformly setose (Fig. 13K); isthmus between antennae about 1.2–1.3 times as broad as diameter of antennal socket (Fig. 13G, K). Antennae short (Fig. 13A, D, G), reaching only behind collum when stretched dorsally, not geniculate, rather strongly clavate due to a particularly enlarged antennomere 6, the latter with a usual, tight, distodorsal group of numerous, bacilliform sensilla; a similar, but smaller, also distodorsal group of sensilla on antennomere 5; antennomere 7 with a smaller distodorsal group of only a few shorter and curved sensilla in front of a tiny mid-dorsal knob.

In width, collum = segment 3 = 4 < 5 < 2 < 6–15 < head; body gradually tapering on segments 16-18 (Fig. 13D–F). Paraterga mostly moderately wide, starting from collum, set rather low (at about upper 1/3 of body height, Fig. 13J), at most small ridges with 2-3 lateral, setigerous knobs, absent from segment 18, caudal corner never produced behind rear tergal margin even in poriferous segments (Fig. 13A–G, M). Ozopores evident, ovoid, dorsolateral, mostly lying closer to lateral edge (Fig. 13M). Collum biconvex, sides (= paraterga) narrowly rounded, dorsal surface with 3 transverse rows of 5+5, 3+3 and 4+4 rather long setae dorsally and 2 similar setae on paraterga (Fig. 13A, D). Each following metatergum with 3+3 or, more rarely, 4+4, similarly long, bacilliform, delicately ribbed setae arranged in 3 transverse regular rows and borne on knobs; sulci between the rows absent (Fig. 13A–G, L, M). Stricture between pro- and metazonae rather deep and narrow, microalveolate like adjacent metazonae (Fig. 13E, L). Limbus very fine, delicately and densely microcrenulate (Fig. 13L, M). Pleurosternal carinae absent (Fig. 13A–C). Epiproct short, conical, truncate, directed caudoventrally; pre-apical papillae evident (Fig. 13C, F, I). Hypoproct subtrapeziform, caudal setigerous papillae moderate and well separated (Fig. 13I).

Sterna without modifications, rather broad and sparsely setose (Fig. 13G, H). Legs short and stout, ca 1.1–1.2 (♂) or 0.9–1.0 (♀) times as long as midbody height; prefemora, femora, postfemora and tibiae clearly incrassate, especially so in ♂ (Fig. 13N), tarsi longest, slender, sphaerotrichomes missing; claws simple, slightly curved; ♂ coxae 2 with very short, membranous, cylindrical gonapophyses (Fig. 13G).

**Figure 14.**
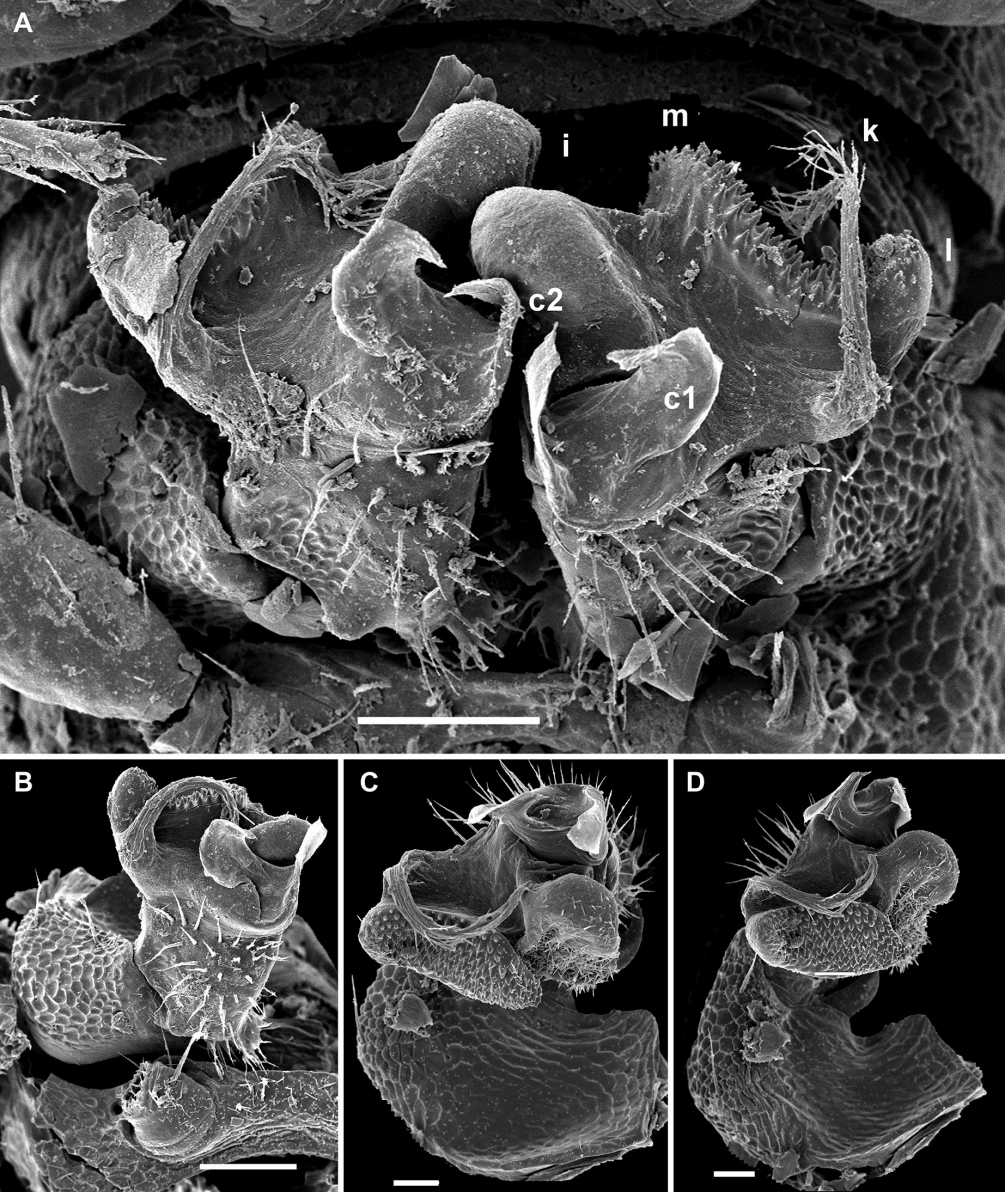
*Helicodesmus anichkini* sp. n., ♂ paratype; **A** both gonopods *in situ*, ventral view **B–D** right gonopod, anteroventral, ventrolateral and lateral views, respectively. Scale bars: **A, B, D** 0.05 mm; **C** 0.02 mm. Designations of gonopod structures in text.

**Figure 15.**
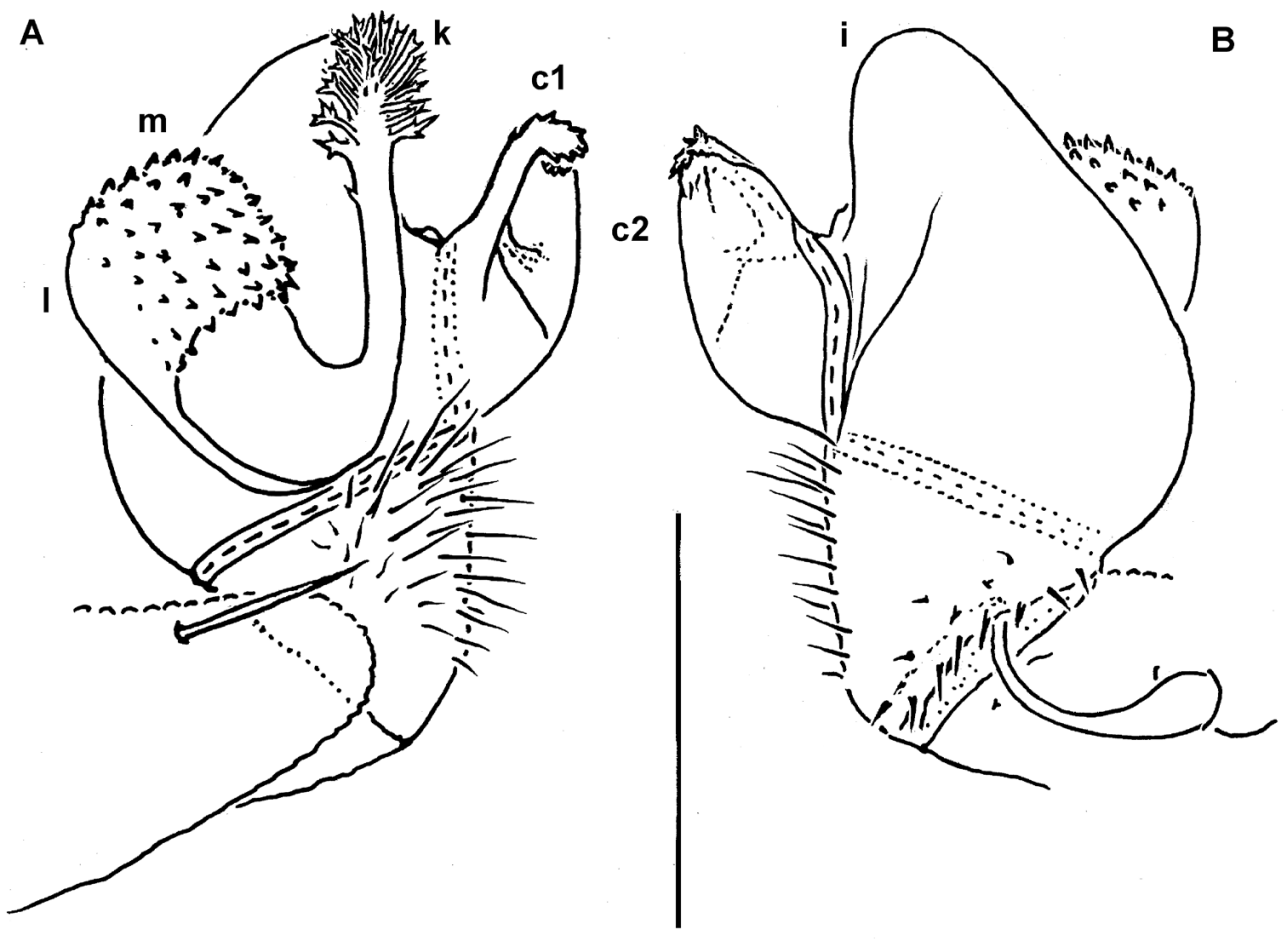
*Helicodesmus anichkini* sp. n., ♂ paratype; **A, B** right gonopod, lateral and mesal views, respectively. Scale bar: 0.1 mm. Designations of gonopod structures in text.

Gonopod aperture transversely oblong-oval, taking up most of ventral part of metazonite 7 (Fig. 14A). Gonopod coxae rather small (gonocoel not deep), sparsely setose and clearly micropapillate laterally (Figs 14, 15); telopodites quite well exposed and unusually transverse, but stout and remarkably complex, very strongly twisted, partly fimbriate(curved ventral projections **c1** and **c2**) or plumose (a long lateral process **k**), with several outgrowths of varying shapes (a stump-shaped, rounded, mesal **i** and 2 indistinctly separated, densely microdentate, also rounded **m** and **l**); prefemoral part about half the height of telopodite, demarcated on lateral (not medial!) side by an oblique seminal groove running further mesad onto a medioventral outgrowth of acropodite to terminate distally at joint base of **c1** and **c2**, with neither a solenomere nor an accessory seminal chamber, nor a pulvillus.

#### Monstrodesmus gen. n.

http://zoobank.org/71C607EC-50F6-49A5-8176-C57F3544EE7A

http://species-id.net/wiki/Monstrodesmus

##### Diagnosis.

19 segments (♂), ♀ unknown; pore formula normal: 5, 7, 9, 10, 12, 13, 15–18; head without modifications; paraterga poorly developed, metaterga with 3 rows of 3+3 or, more rarely, 4+4, long, bacilliform setae (regardless of lateral setae); gonopod coxae large (gonocoel quite deep); telopodites rather well exposed, tripartite, without evidence of torsion, elongate, moderately curved caudad, subcontiguous medially and held parallel to each other; acropodites lying distal to prefemoral parts much longer than and coaxial with the latter; solenomere (**sl**), or endomere, a strong, simple, frontal branch about as long as a lateral exomere (**ex**); a very long, flagelliform, mesal branch (**fl**) at base of both **sl** and **ex**; seminal groove (**sg**) running entirely along mesal side of **sl** to terminate on top with neither an accessory seminal chamber nor a pulvillus.

##### Name.

To emphasize the monstrously long flagellum of the gonopod, masculine.

##### Type species.

*Monstrodesmus flagellifer* sp. n., by present designation.

##### Remarks.

This new genus seems to be particularly similar to *Topalodesmus* Golovatch, 1988, monobasic, from the Himalayas of India (Golovatch 1988b). Indeed, both share basically the same gonopod conformation: coxae quite massive; gonocel rather deep, but telopodites clearly exposed, deeply tripartite, untwisted, curved caudad, subcontiguous medially and held parallel to main axis; seminal groove running entirely mesally along a very strong frontal endomere branch (= solenomere, **sl**). However, the differences are definitely significant enough to keep these two genera separate. Unlike *Topalodesmus* which shows 20 segments in both sexes, the gonopod coxa in *Monstrodesmus* gen. n. is devoid of a frontal process, the exomere (**ex**) prominent (vs vestigial), the caudomesal branch (**fl**) unusually long and flagelliform (vs thick and unciform) while the solenomere (**sl**) a very long, strong, frontal branch.

#### Monstrodesmus flagellifer sp. n.

http://zoobank.org/46A8E604-6546-481A-A138-25C47E2323A6

http://species-id.net/wiki/Monstrodesmus_flagellifer

Figs 16 – 18

##### Type material.

Holotype ♂ (MNHN JC 361), Vietnam, Dongnai Prov., Cat Tien National Park, 107°10'–107°34'E, 11°21'–11°48'N, lowland tropical forest, litter and topsoil, 01.06.2005, leg. A. Anichkin.

Paratype: 1 ♂ (SEM), same locality, together with holotype.

##### Name.

The species epithet, a noun in apposition, is to emphasize the remarkably long gonopod flagellum.

##### Description.

Length of adults ca 4.5 mm, width of midbody pro- and metazonae 0.33 and 0.4 mm (♂). Coloration in alcohol uniformly pallid, tegument often nearly translucent.

**Figure 16.**
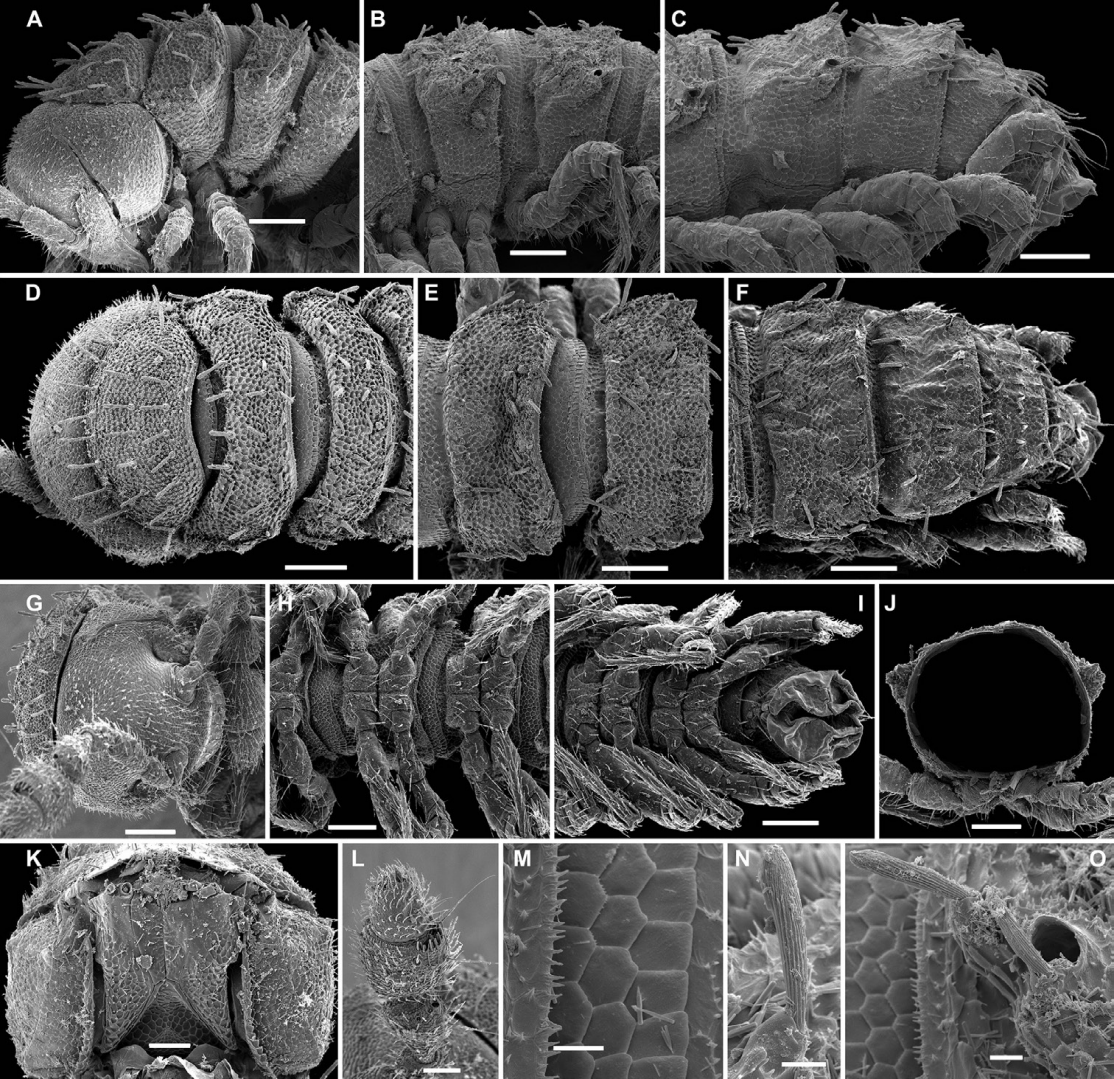
*Monstrodesmus flagellifer* sp. n., ♂ paratype; **A, D** anterior body part, lateral and dorsal views, respectively **B, E, H** midbody segments, lateral, dorsal and ventral views, respectively **C, F, I** posterior body part, lateral, dorsal and ventral views, respectively **G, K** head, frontal and ventral views, respectively **J** cross-section of a midbody segment, caudal view **L** antennomeres 4-8, sublateral view; tergal seta **M** tegument structure, limbus and stricture region **N** tergal seta, lateral view **O** ozopore region of a midbody segment, sublateral view. Scale bars: **A–J** 0.1 mm; **K, L** 0.05 mm; **M–O** 0.01 mm.

Body with 19 segments (♂). Tegument dull, texture of nearly entire body delicately alveolate to scaly, only sterna nearly smooth (Fig. 16A–I). Head very densely and finely pilose; epicranial suture highly superficial; genae squarish (Fig. 16A, D, G); gnathochilarium rather broad, sparsely and uniformly setose (Fig. 16K); isthmus between antennae about 1.2 times as broad as diameter of antennal socket (Fig. 16G). Antennae short (Fig. 16G, L), reaching only behind collum when stretched dorsally, not geniculate, rather strongly clavate due to a particularly enlarged antennomere 6, the latter with a usual, tight, distodorsal group of numerous, bacilliform sensilla; a similar, but smaller, also distodorsal group of sensilla on antennomere 5; antennomere 7 with a smaller distodorsal group of only a few shorter and curved sensilla in front of a tiny mid-dorsal knob.

In width, collum < segment 3 = 4 < head = 2 < 5-15; thereafter body gradually tapering (Fig. 16D–F). Paraterga mostly moderate, starting from collum, set rather low (at about upper 1/3 of body height, Fig. 16J), at most small ridges with 2-3 lateral, setigerous knobs, absent from segment 18, caudal corner never produced behind rear tergal margin even in poriferous segments (Fig. 16A–I, O). Ozopores evident, ovoid, dorsolateral, mostly lying closer to lateral edge (Fig. 16B, C, E, F, O). Collum subovoid, sides (= paraterga) well rounded, dorsal surface with 3 transverse rows of 4+4, 3+3 and 3+3 rather long setae dorsally and 2 similar setae on paraterga (Fig. 16A, D). Each following metatergum with 3+3 or, more rarely, 4+4, similarly long, bacilliform, delicately ribbed setae arranged in 3 transverse regular rows and borne on knobs; sulci between the rows superficial (Fig. 16A–F). Stricture between pro- and metazonae rather deep and narrow, microalveolate like adjacent metazonae (Fig. 16A–F, M, O). Limbus very fine, delicately and densely microspiculate (Fig. 16M, O). Pleurosternal carinae absent (Fig. 16A–C). Epiproct short, conical, truncate, directed caudoventrally; pre-apical papillae small (Fig. 16C, F, I). Hypoproct subtrapeziform, caudal setigerous papillae moderate and well separated (Fig. 16I).

**Figure 17.**
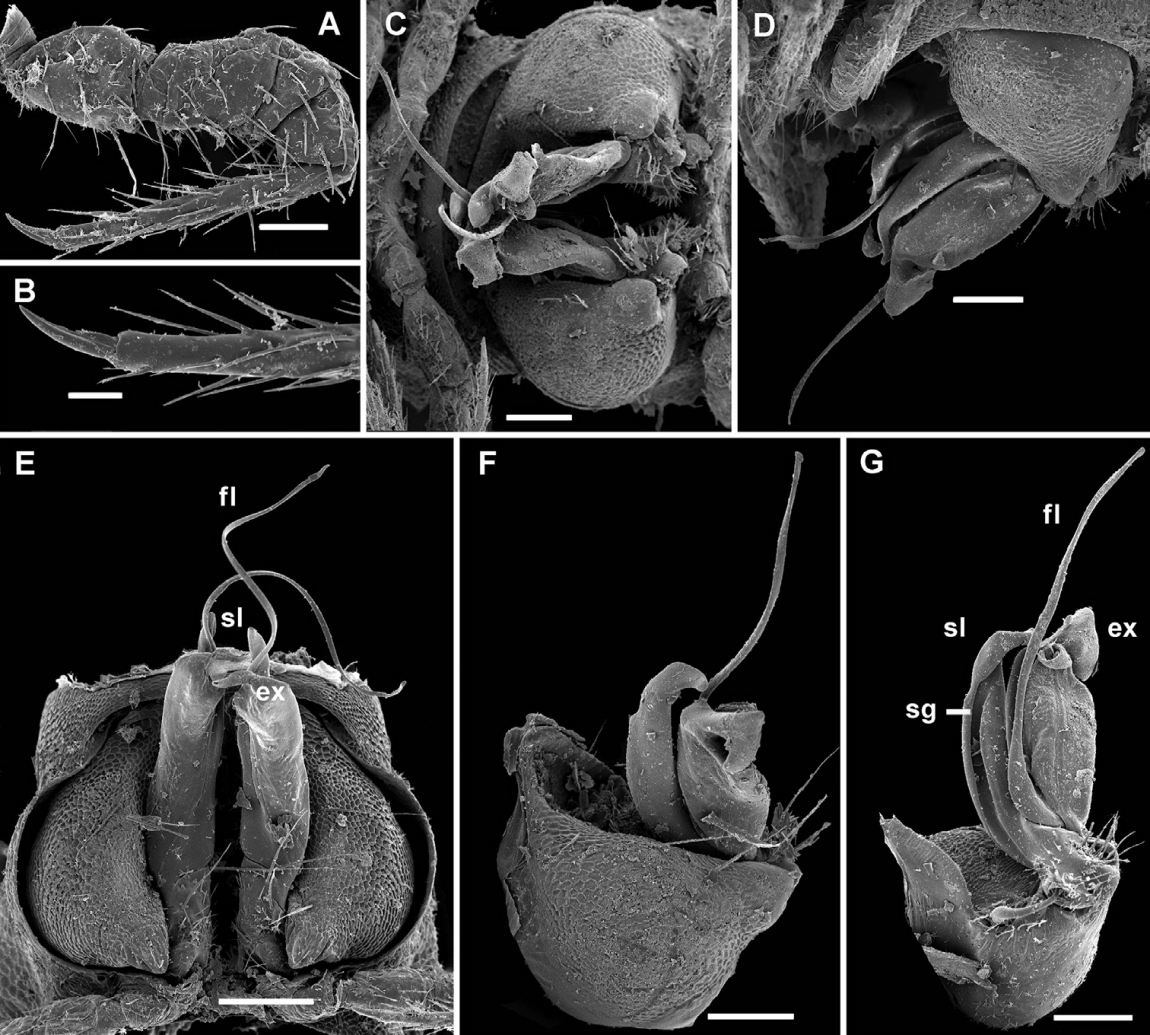
*Monstrodesmus flagellifer* sp. n., ♂ paratype; **A** midbody leg, lateral view **B** claw of a midbody leg **C–E** both gonopods *in situ*, ventral, lateral and ventrocaudal views, respectively **F** right gonopod, lateral view **G** left gonopod, mesal view. Scale bars: **C–G** 0.1 mm; **A** 0.05 mm; **B** 0.02 mm. Designations of gonopod structures in text.

Sterna without modifications, rather broad and sparsely setose (Fig. 16H, I). Legs short and stout, ca 1.1-1.2 times as long as midbody height (♂); prefemora, femora, postfemora and tibiae clearly incrassate (Fig. 17A), tarsi longest, slender, sphaerotrichomes missing; claws simple, slightly curved (Fig. 17B); coxae 2 with very short, membranous, cylindrical gonapophyses.

**Figure 18.**
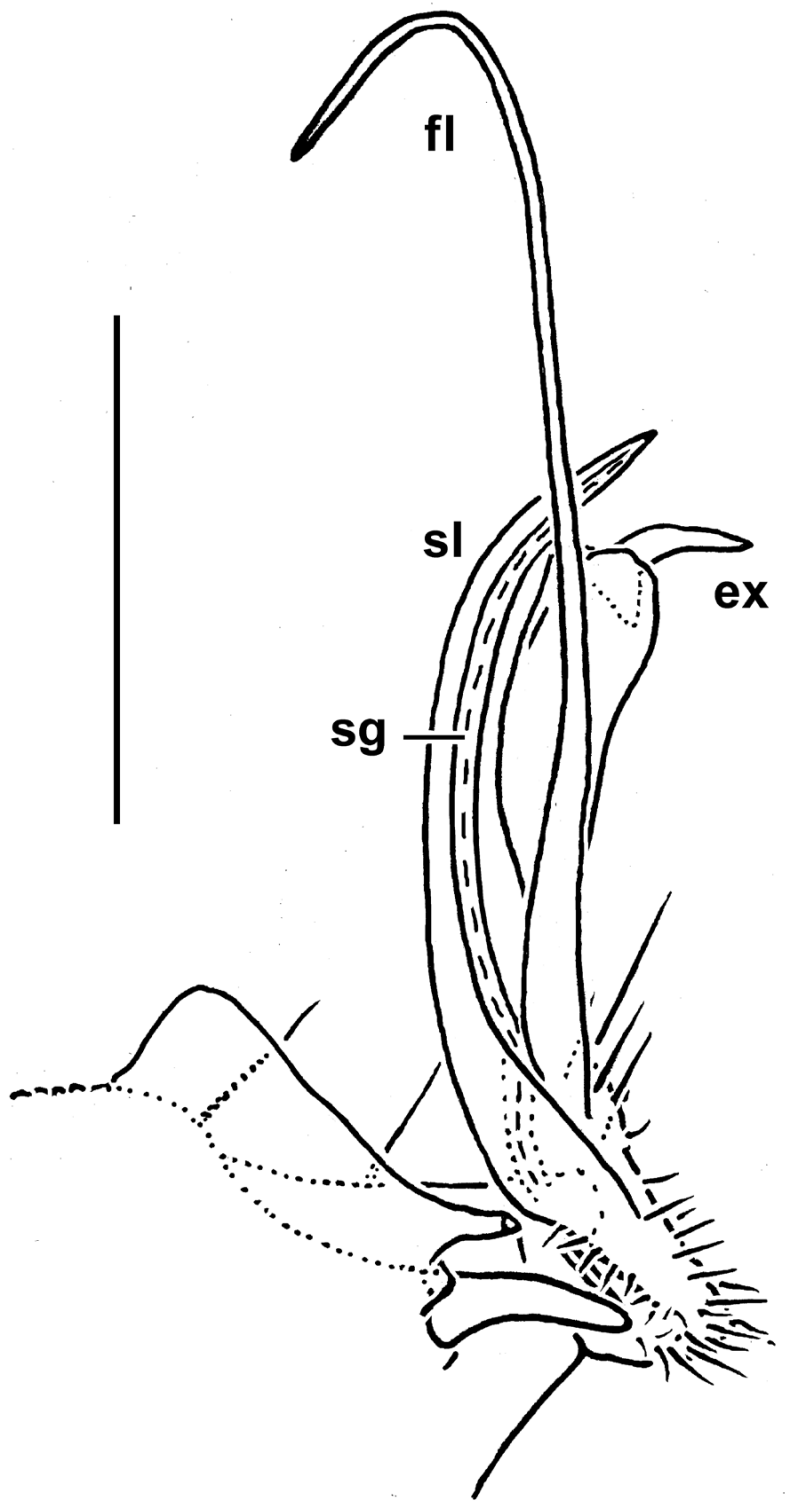
*Monstrodesmus flagellifer* sp. n., ♂ holotype, right gonopod, mesal view. Scale bar: 0.2 mm. Designations of gonopod structures in text.

Gonopod aperture transversely obcordate, taking up most of ventral part of metazonite 7 (Fig. 17C, E). Gonopod coxae large (gonocoel rather deep), with 2 long setae and clearly micropapillate laterally, but without any frontal outgrowths (Fig. 17C–G, 18); telopodites quite well exposed, not twisted, tripartite, elongate, moderately curved caudad, subcontiguous medially and held parallel to each other; acropodites lying distal to prefemoral parts much longer than and coaxial with the latter; solenomere (**sl**) (= endomere) a strong, simple, frontal branch about as long as an apically bifid lateral exomere (**ex**); an extremely long, flagelliform, mesal branch (**fl**) at base of both **sl** and **ex**; seminal groove (**sg**) running entirely along mesal side of **sl** to terminate on top with neither an accessory seminal chamber nor a pulvillus.

### A key to genera and species of Trichopolydesmidae currently known from Vietnam/Indochina, based mainly on male characters

Since brief diagnoses of all previously known Oriental trichopolydesmid genera are given above, and their scopes and statuses also considered, below we only present a key to the new genera and species, the first to be reported from Indochina in general and from Vietnam in particular.

**Table:** 

1	Ozopores totally missing (Figs 3A–F, 5A–F, 7A–F)	*Aporodesmella* gen. n., 2
–	Pore formula normal	4
2	Paraterga strongly developed (Fig. 7A–F). ♂ head with an evident, round, very finely pilose, central hump above antennae (Fig. 7D, G). Antennomere 6 unmodified (Fig. 7J). Gonopods as in Fig. 8C–F	*Aporodesmella tergalis* sp. n.
–	Paraterga poorly developed to missing (Figs 3A–F,5A–F). ♂ head unmodified. Antennomere 6 with a peculiar dorsoparabasal stump (**s**) (Figs 3J, 4A, 5G). Gonopods different	3
3	Solenophore (**sph**) unusually short and axe-shaped, solenomere (**sl**) simple and lanceolate (Fig. 4D–G)	*Aporodesmella securiformis* sp. n.
–	Gonopod tip more elaborate, both **sph** and **sl** different (Fig. 6B, C)	*Aporodesmella similis* sp. n.
4	♂ head with an evident, round, central hump above antennae (Fig. 11A, D, G). Gonopod telopodites clearly geniculate due to a midway cingulum (**c**), distal half directed laterad, with an unusually strongly developed hairy pulvillus (**p**) (Figs 12D–G)	*Gonatodesmus* gen. n. (*Gonatodesmus communicans* sp. n.)
–	♂ head devoid of a hump. Gonopod telopodites non-geniculate, at most strongly, but regularly curved, distal parts never directed laterad; a hairy pulvillus absent	5
5	Head in both sexes somewhat flattened dorsoventrally (Fig. 9A, D, G). Gonopod telopodites long and slender, semi-circular, *in situ* crossing medially, bifid subapically (Fig. 10C–E)	*Deharvengius* gen. n. (*Deharvengius bedosae* sp. n.)
–	Head clearly more convex dorsally. Gonopod telopodites far more elaborate	6
6	Gonopods especially complex, unusually strongly twisted, *in situ* clearly transverse, devoid of a solenomere (Figs 14, 15)	*Helicodesmus* gen. n. (*Helicodesmus anichkini* sp. n.)
–	Gonopod telopodites held parallel to main axis, each tripartite, including a remarkably long flagellum (**fl**) (Figs 17C–G, 18)	*Monstrodesmus* gen. n. (*Monstrodesmus flagellifer* sp. n.)

## A brief discussion and conclusions

As observed elsewhere (Golovatch et al. 2013), most of the Oriental Trichopolydesmidae seem to be moderately advanced. Only some show complex and strongly shortened gonopod telopodites deeply sunken inside the gonocoel formed by enlarged coxae, presumably a more advanced condition. In the majority of genera and species, however, the telopodites are strongly exposed, a more primitive situation. The orientation of the prefemoral part also varies, but it tends to be held parallel, not transversely, to the main axis of the body, a condition typical of the superfamily Polydesmoidea.

*Gonatodesmus* gen. n., in which the gonopods are considerably elongate, geniculate at about midway while their distal halves are directed laterad, provides a kind of transition or evolutionary bridge between Trichopolydesmidae and Opisotretidae, thus reinforcing the assignment of these two families to the single superfamily Trichopolydesmoidea. This is in contrast to the much more frequent condition of the gonotelopodites as observed in Trichopolydesmidae and most other groups of Polydesmida, which either cross mesally or are held parallel to each other. Based on this example, the Opisotretidae might well be viewed as a direct and disjunct derivative of the more basal Trichopolydesmidae, with their gonotelopodite growing increasingly elongate and orientated laterally (Golovatch et al. 2013).

There can be no doubt that many more species of Trichopolydesmidae await discovery and description. Representatives of this family might seem to be rare, but it is very likely that they are seriously under-collected due to their very small size and perhaps also to seasonality. Thus, most of the adults have been taken during or shortly after the rainy season (May to November), when the animals are surface-active. During the dry season, like most other Diplopoda in lowland tropical forests of Vietnam, the very small trichopolydesmoids seem to be dormant (Golovatch et al. 2011). At least in Vietnam, sympatry, even syntopy, of several (≥ three) different trichopolydesmid species also seems to be common.

## Supplementary Material

XML Treatment for Trichopolydesmidae

XML Treatment for Assamodesmus

XML Treatment for Cocacolaria

XML Treatment for Coonoorophilus

XML Treatment for Hingstonia

XML Treatment for Kukkalodesmus

XML Treatment for Magidesmus

XML Treatment for Mastodesmus

XML Treatment for Nasodesmus

XML Treatment for Ootacadesmus

XML Treatment for Peronorchus

XML Treatment for Pseudosphaeroparia

XML Treatment for Sholaphilus

XML Treatment for Topalodesmus

XML Treatment for Cocacolaria hauseri

XML Treatment for Aporodesmella

XML Treatment for Aporodesmella securiformis

XML Treatment for Aporodesmella similis

XML Treatment for Aporodesmella tergalis

XML Treatment for Deharvengius

XML Treatment for Deharvengius bedosae

XML Treatment for Gonatodesmus

XML Treatment for Gonatodesmus communicans

XML Treatment for Helicodesmus

XML Treatment for Helicodesmus anichkini

XML Treatment for Monstrodesmus

XML Treatment for Monstrodesmus flagellifer

## References

[B1] Attems C (1907) Javanische Myriopoden gesammelt von Direktor K. Kraepelin im Jahre 1903. Mitteilungen aus dem Naturhistorischen Museum Hamburg 24: 77–122

[B2] Bouchet F Le Guayder H Pascal O (2009) The SANTO 2006 Global Biodiversity Survey: An attempt to reconcile the pace of taxonomy and conservation. Zoosystema 31(3): 401–406. doi: 10.5252/z2009n3a0

[B3] Bouchet F Le Guayder H Pascal O (Eds) (2011) The natural history of Santo. Patrimoines Naturels 70 Muséum national d’Histoire naturelle, Paris, 572 pp

[B4] Bouchet F Le Guayder H Pascal O (2012) The altruism of biodiversity exploration expeditions. Zoosystema 34(2): 193–202. doi: 10.5252/z2012n2a0

[B5] Carl J (1911) Drei neue Diplopoden des Genfer Museums. Revue suisse de Zoologie 19(16): 397–407

[B6] Carl J (1932) Diplopoden aus Süd-Indien und Ceylon. 1. Teil. Polydesmoidea. Revue suisse de Zoologie 35(17): 411–529

[B7] Carl J (1935) Polydesmiden gesammelt von Major R. W. Hingston auf der III. Everest-Expedition, 1924. Revue suisse de Zoologie 42(10): 325–340

[B8] Cook OF (1896) A new diplopod fauna in Liberia. The American Naturalist 30: 413–420

[B9] Golovatch SI (1986) Diplopoda from the Nepal Himalayas: Polydesmidae, Fuhrmannodesmidae. Senckenbergiana biologica 66(4/6): 345–369 [for 1985].

[B10] Golovatch SI (1987) Diplopoda from the Nepal Himalayas. Opisotretidae, additional Polydesmidae and Fuhrmannodesmidae. Courier Forschungsinstitut Senckenberg 93: 203–217

[B11] Golovatch SI (1988a) On the first Polydesmidae, Opisotretidae and Fuhrmannodesmidae from Bhutan (Diplopoda, Polydesmida). Entomologica Basiliensia 12: 15–48

[B12] Golovatch SI (1988b) On three remarkable genera of Polydesmoidea (Diplopoda: Polydesmida) from the Himalayas of India. Folia Entomologica Hungarica 49: 41–47

[B13] Golovatch SI (1990) Diplopoda from the Nepal Himalayas. Several additional Polydesmidae and Fuhrmannodesmidae (Polydesmida). Spixiana 13: 237–252

[B14] Golovatch SI (1992) Review of the Neotropical fauna of Fuhrmannodesmidae, with the description of four new species from near Manaus, Central Amazonia, Brazil (Diplopoda, Polydesmida). Amazoniana 12(2): 207–226

[B15] Golovatch SI (1994) Further new Fuhrmannodesmidae from the environs of Manaus, Central Amazonia, Brazil, with a revision of *Cryptogonodesmus* Silvestri, 1898 (Diplopoda, Polydesmida). Amazoniana 13(1/2): 131–161

[B16] Golovatch SI (2011) The millipede genus *Caucasodesmus* Golovatch, 1985, with the description of a new species from the Crimea, Ukraine (Polydesmida, Diplopoda, Trichopolydesmidae). ZooKeys 93: 1–8. doi: 10.3897/zookeys.93.1159 21594076 10.3897/zookeys.93.1159 PMC3095180

[B17] Golovatch SI (2013) A reclassification of the millipede superfamily Trichopolydesmoidea, with descriptions of two new species from the Aegean region (Diplopoda, Polydesmida). ZooKeys 340: 63–78. doi: 10.3897/zookeys.340.6295 24146592 10.3897/zookeys.340.6295 PMC3800799

[B18] Golovatch SI Geoffroy JJ Mauriès JP VandenSpiegel D (2009a) Review of the millipede family Haplodesmidae Cook, 1895, with descriptions of some new or poorly-known species (Diplopoda, Polydesmida). ZooKeys 7: 1–53. doi: 10.3897/zookeys.7.117 10.3897/zookeys.505.9862 PMC4453233 26052236

[B19] Golovatch SI Geoffroy JJ Mauriès JP VandenSpiegel D (2009b) Review of the millipede genus *Eutrichodesmus* Silvestri, 1910 (Diplopoda, Polydesmida, Haplodesmidae), with descriptions of new species. ZooKeys 12: 1–46. doi: 10.3897/zookeys.12.167 10.3897/zookeys.505.9862 PMC4453233 26052236

[B20] Golovatch SI Geoffroy JJ Stoev P VandenSpiegel D (2013) Review of the millipede family Opisotretidae (Diplopoda, Polydesmida), with descriptions of new species. ZooKeys 302: 13–77. doi: 10.3897/zookeys.302.5357 23794898 10.3897/zookeys.302.5357 PMC3689142

[B21] Golovatch SI Mikhaljova EV Korsós Z Chang HW (2010) The millipede family Haplodesmidae (Diplopoda, Polydesmida) recorded in Taiwan for the first time, with the description of a new species. Tropical Natural History 10(1): 27–36

[B22] Golovatch SI Tiunov AV Anichkin AE (2011) [Millipedes (Diplopoda)]. In: Tiunov AV (Ed) Structure and functions of soil communities of a monsoon tropical forest (Cat Tien National Park, southern Vietnam). KMK Scientific Press, Moscow, 76–90 [In Russian, with an English abstract]

[B23] Golovatch SI Liu WX Li YB Geoffroy JJ (2012) One new and two little-known species of the millipede family Polydesmidae from southern China (Diplopoda: Polydesmida). Arthropoda Selecta 21(2): 131–136

[B24] Hoffman RL (1980) Classification of the Diplopoda. Muséum d’histoire naturelle, Genève, 237 pp [for 1979].

[B25] Hoffman RL (1982) Diplopoda. In: Parker SP (Ed) Synopsis and classification of living organisms, 2 McGraw-Hill, New York, St Louis, 689–774

[B26] Hoffman RL (1987) A new genus and species of polydesmoid millipede from New Ireland. Revue suisse de Zoologie 94(1): 35–39

[B27] Humbert A (1865) Essai sur les myriapodes de Ceylan. Mémoires de la Société de Physique et d’Histoire naturelle de Genève 18(1): 1–62

[B28] Jeekel CAW (1970) Nomenclator generum et familiarum Diplopodorum. A list of genus and family-group names in the class Diplopoda from the 10^th^ edition of Linnaeus, 1758, to the end of 1957. Monografieën van de Nederlandse Entomologische Vereiniging 5: i-xii, 1–412

[B29] Manfredi P (1955) Un nouveau polydesmien cavernicole de l’Assam (Inde). Notes biospéologiques 9: 141–144 [for 1954]

[B30] Mauriès JP (1983) Le genre *Galliocookia* Ribaut, 1954. Deux especes nouvelles des grottes de l’Ardeche et du Gard (Myriapoda, Diplopoda, Polydesmida). Bulletin de la Société d’Histoire naturelle de Toulouse 119: 103–110

[B31] Mauriès JP Geoffroy JJ (1999) Les diplopodes édaphiques et souterrains de l’Ile Maurice (Myriapoda, Diplopoda). Revue suisse de Zoologie 106(1): 69–79

[B32] Mauriès JP Heymer A (1996) Nouveaux micropolydesmides d’Afrique centrale: essai de rassemblement pour une révision du genre *Sphaeroparia* (Diplopoda, Polydesmida, Fuhrmannodesmidae). Bulletin du Muséum national d’Histoire naturelle, série 4, 18A(1–2): 165–184

[B33] Shear WA (1986) Identity of the Indian cave milliped *Typhlopymaeosoma hazeltonae* Turk (Polydesmida, Opisotretidae?): another case of ordinal misplacement in the Diplopoda. Myriapodologica 2(2): 7–12

[B34] Simonsen Å (1990) Phylogeny and biogeography of the millipede order Polydesmida, with special emphasis on the suborder Polydesmidea. Museum of Zoology, University of Bergen, Bergen, 114 pp

[B35] Turk FA (1945) On two diplopods of the family Vanhoeffeniidae from Indian caves, with the description of a new genus. Annals and Magazine of Natural History (11) 12: 38–42. doi: 10.1080/00222934508527486

[B36] Turk FA (1972) A new blind millipede (*Typhlopymeosoma hazeltonae* n. gen., n. sp.) from a Himalayan cave with notes on its zoogeographical significance. Transactions of the Cave Research Group of Great Britain 14(4): 195–198

